# Perovskite quantum dots in cancer diagnosis and therapy: from synthesis to biomedical applications

**DOI:** 10.1039/d5ra08157b

**Published:** 2025-12-01

**Authors:** Mohammad Abushuhel, G. PadmaPriya, Shaker Al-Hasnaawei, Subhashree Ray, Kattela Chennakesavulu, Renu Sharma, Ashish Singh Chauhan, Hadi Noorizadeh, Mosstafa Kazemi

**Affiliations:** a Faculty of Allied Medical Sciences, Hourani Center for Applied Scientific Research, Al-Ahliyya Amman University Amman Jordan; b Department of Chemistry and Biochemistry, School of Sciences, JAIN (Deemed to be University) Bangalore Karnataka India; c College of Pharmacy, Islamic University Najaf Iraq; d Department of Medical Analysis, Medical Laboratory Technique College, Islamic University of Al Diwaniyah Al Diwaniyah Iraq; e Department of Biochemistry, IMS and SUM Hospital, Siksha ‘O’ Anusandhan Bhubaneswar Odisha-751003 India; f Department of Chemistry, Sathyabama Institute of Science and Technology Chennai Tamil Nadu India; g Department of Chemistry, University Institute of Sciences, Chandigarh University Mohali Punjab India; h Uttaranchal Institute of Pharmaceutical Sciences, Division of Research and Innovation, Uttaranchal University Dehradun Uttarakhand India; i Young Researchers and Elite Club, Tehran Branch, Islamic Azad University Tehran Iran hadinoorizadehacademic@gmail.com

## Abstract

Perovskite quantum dots (PQDs) have emerged as a new generation of semiconductor nanomaterials with outstanding potential in oncology. Their unique optoelectronic features—including high photoluminescence quantum yields, tunable emission, and efficient charge transport—position them as superior candidates compared to conventional quantum dots. This review presents an integrated overview of PQDs, starting from their synthesis methodologies and structural–optoelectronic characteristics to their biocompatibility and biomedical applications. Special attention is paid to surface modification strategies, such as silica encapsulation, polymer coatings, hybrid nanostructures, and biomimetic approaches, which enhance aqueous stability, mitigate toxicity, and enable targeted delivery. Furthermore, the applications of PQDs in cancer diagnostics and therapy are highlighted, covering fluorescence and multimodal imaging, biosensing of tumor biomarkers, and advanced therapeutic modalities including photodynamic, photothermal, and integrated theranostic platforms. This review is among the first to systematically link PQD synthesis and property engineering with practical oncological applications. By addressing current limitations while outlining biomedical opportunities, this work emphasizes the promise of PQDs as versatile tools for next-generation cancer diagnosis and therapy.

## Abstract 2

Review of PQDs: synthesis, properties, & biomedical uses. Focus on surface coatings to improve stability, reduce toxicity, and enable targeted delivery.

## Graphical abstract

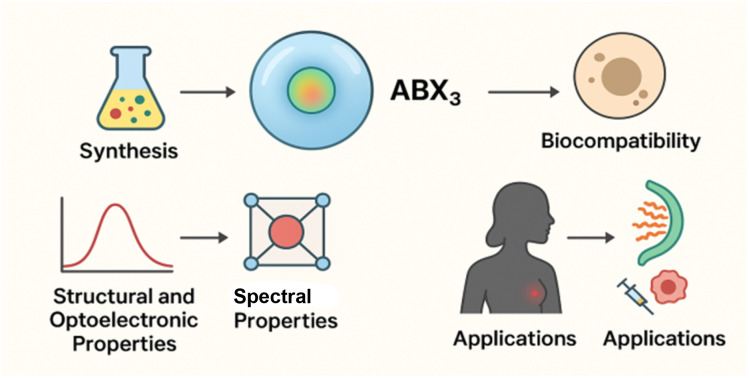

## 1. Introduction

Cancer continues to be one of the most devastating health challenges globally, accounting for millions of deaths each year despite remarkable progress in early detection, therapeutic technologies, and clinical management.^1–4^ Traditional treatment modalities such as chemotherapy,^5,6^ radiotherapy,^7,8^ and surgery,^9,10^ although widely used, often suffer from limitations including lack of specificity, systemic toxicity, and reduced efficacy against metastatic and drug-resistant tumors. These limitations have fueled the search for new approaches that allow precise diagnosis, real-time monitoring, and targeted therapy with minimal side effects.^11,12^ Nanotechnology, with its capacity to manipulate matter at the molecular and nanoscale, has emerged as a powerful tool in this endeavor, providing unique opportunities for designing advanced diagnostic and therapeutic platforms.^13–15^ Among nanomaterials, quantum dots (QDs) have been investigated extensively due to their distinctive optical and electronic properties. Their size-tunable emission, broad absorption, and strong photostability make them attractive for biomedical imaging and sensing.^16–18^ However, conventional QDs such as CdSe/ZnS or InP face challenges including broad emission spectra, limited quantum yields, and in many cases, significant toxicity due to heavy metal content. These drawbacks have constrained their widespread use in biomedical contexts and motivated the exploration of alternative semiconductor nanomaterials with improved performance and biocompatibility.^19–21^

In recent years, perovskite PQDs have gained considerable attention as a new generation of nanomaterials with properties that surpass many limitations of traditional QDs.^22–24^ With the general formula ABX_3_, PQDs exhibit exceptional photoluminescence quantum yields, narrow emission linewidths, defect tolerance, and facile tunability across the visible and near-infrared range.^25,26^ Their unique ionic–covalent bonding nature, large exciton Bohr radius, and efficient charge transport further enhance their suitability for optoelectronic and biomedical applications. The rapid progress in PQD research has led to their successful implementation in solar cells, light-emitting diodes, lasers, and most recently, in biomedical imaging and cancer therapy.^27,28^ The integration of PQDs into oncology is particularly intriguing. Their high brightness and narrow emission enable precise visualization of biological processes at the cellular and tissue levels.^29^ Their tunability across a wide spectral range supports multiplexed imaging, while their strong absorption cross-section allows deep-tissue penetration through multi-photon excitation. Moreover, PQDs possess physicochemical versatility that makes them amenable to surface modification and functionalization with biomolecules such as peptides, antibodies, or nucleic acids, which are critical for achieving specificity in cancer targeting.^30–32^ These features collectively position PQDs as promising candidates for next-generation platforms in cancer diagnosis, biosensing, and therapy.^33,34^

Despite these advantages, several critical challenges remain. PQDs, particularly those containing lead, raise concerns about toxicity and long-term biosafety. Their intrinsic instability in aqueous and physiological environments further complicates their direct biomedical use.^35,36^ Exposure to moisture, oxygen, light, or heat can result in degradation, halide segregation, and loss of luminescence. These issues necessitate robust strategies for surface passivation, encapsulation, and development of lead-free alternatives to ensure biocompatibility and stability under physiological conditions.^37,38^ Another challenge relates to the scalability and reproducibility of PQD synthesis, as biomedical applications require consistent and standardized materials that can meet regulatory requirements for clinical translation.^39,40^ Addressing these challenges requires interdisciplinary collaboration between chemists, material scientists, biomedical engineers, and oncologists. Efforts are increasingly focused on the design of environmentally friendly synthesis approaches, the development of innovative surface engineering techniques, and the exploration of novel compositions that maintain high optical performance while minimizing toxicity.^41^ Simultaneously, there is growing emphasis on integrating PQDs into multifunctional nanoplatforms capable of combining diagnostic and therapeutic roles—so-called theranostic systems—that hold promise for personalized and precision medicine.^42,43^

The rapidly expanding literature on PQDs spans a wide spectrum of disciplines, from fundamental physics and chemistry to applied biomedical research. Yet, despite this growth, there remains a lack of comprehensive reviews that systematically connect the basic material properties, synthesis methods, biocompatibility considerations, and cancer-related applications of PQDs. Most reports either emphasize optoelectronic applications or provide fragmented discussions on biomedical aspects without an integrative framework.^44–46^

This review addresses this gap by providing a systematic and integrative overview of PQDs, bridging their fundamental structural and optoelectronic properties with their biomedical applications. Beginning with an analysis of their tunable optical characteristics and conventional and innovative synthesis strategies, the article delves into biocompatibility challenges, advanced surface modification techniques, and their emerging roles in cancer imaging, biomarker detection, and therapeutic modalities, including photodynamic, photothermal, and theranostic platforms. Unlike prior reviews that predominantly focused on optoelectronic applications,^37,45–50^ this work is the first to explicitly situate PQDs at the intersection of materials science and oncology, offering a comprehensive perspective that connects fundamental chemistry with practical clinical outcomes. By highlighting current achievements, addressing critical challenges for clinical translation, and proposing future research directions, this review aims to stimulate cross-disciplinary collaboration and establish PQDs as reliable tools for next-generation cancer care.

## 2. Properties and synthesis of perovskite quantum dots

PQDs represent a groundbreaking class of semiconductor nanomaterials that have revolutionized fields ranging from optoelectronics to biomedicine. Characterized by the general chemical formula ABX_3_—where A is typically a monovalent organic or inorganic cation such as methylammonium (MA^+^), formamidinium (FA^+^), or cesium (Cs^+^); B is a divalent metal cation like lead (Pb^2+^), tin (Sn^2+^), or germanium (Ge^2+^); and X is a halide anion (Cl^−^, Br^−^, or I^−^)—PQDs exhibit pronounced quantum confinement effects due to their nanoscale dimensions, often confined to 2–15 nm.^51–53^ This confinement not only discretizes the energy levels but also enables precise tuning of optoelectronic properties, making PQDs exceptionally versatile for applications in cancer diagnosis and therapy. Unlike traditional quantum dots, PQDs combine high ionic character with covalent bonding, leading to unique defect tolerance and superior luminescence efficiency.^54,55^ This section provides an in-depth exploration of their fundamental properties, synthesis methodologies, innovative strategies, and a comparative analysis with other quantum dot systems, highlighting why PQDs are poised to outperform conventional alternatives in biomedical contexts.

### 2.1. Fundamental optical, electronic, and structural properties

The optical prowess of PQDs is rooted in their quantum-confined electronic structure, where the particle size dictates the bandgap energy (*E*_g_) *via* the effective mass approximation:^56^

1 *E*_g_ = *E*_bulk_ + (ℏ^2^π^2^)/(2*µr*^2^)

where *µ* is the reduced exciton mass and *r* is the nanocrystal radius. For lead halide perovskites, the Bohr exciton radius is relatively large (*e.g.*, ∼7 nm for CsPbBr_3_), allowing strong confinement even in larger nanocrystals compared to II–VI QDs like CdSe (∼5 nm). This results in tunable emission from ultraviolet to near-infrared by varying halide composition: CsPbCl_3_ emits blue (∼410 nm), CsPbBr_3_ green (∼510 nm), and CsPbI_3_ red (∼690 nm). Mixed-halide systems (*e.g.*, CsPb(Br/I)_3_) enable continuous spectral tuning, with photoluminescence quantum yields (PLQYs) routinely exceeding 90% due to minimal non-radiative recombination. The narrow emission linewidths (FWHM ∼12–40 nm) arise from homogeneous size distributions and reduced inhomogeneous broadening, far superior to organic dyes (FWHM >50 nm), facilitating high-resolution multiplexed imaging in cancer diagnostics.^57,58^

Electronically, PQDs display ambipolar charge carrier transport with electron and hole mobilities of 10–450 cm^2^ V^−1^ s^−1^, attributed to the low effective masses (
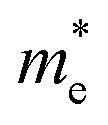
 ∼ 0.1–0.2*m*_0_) and long carrier diffusion lengths (>1 µm). The dielectric constant (*ε*_r_ ∼20–30) screens excitons effectively, yielding binding energies of 15–75 meV, which stabilize excitonic states at physiological temperatures.^59^ This enables efficient energy transfer processes like Förster resonance energy transfer (FRET) in bioimaging probes. Structurally, PQDs adopt a cubic (*Pm*3̄*m*) perovskite lattice at room temperature, with BX_6_ octahedra forming a 3D framework stabilized by A-site cations. The tolerance factor (*t*) and octahedral factor (*µ*) govern phase stability: ideal cubic phases occur for *t* ∼ 0.9–1.0 and *µ* ∼ 0.4–0.7. Deviations induce tilting, leading to orthorhombic or tetragonal polymorphs, which can alter bandgap by 0.1–0.3 eV and influence charge trapping.^60^

**Fig. 1:**
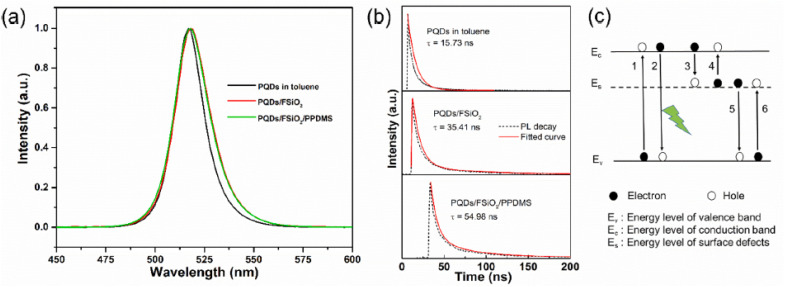
(a) PL spectra and (b) time-resolved PL decay curves (*τ* = 17.53 ns in toluene → 35.41 ns in FSiO_2_ → 54.98 ns in FSiO_2_/PPDMS foam) of CsPbBr_3_ QDs, showing effective trap passivation and photon recycling. (c) Energy-level diagram of radiative and non-radiative pathways. The PPDMS foam matrix preserves optical performance while providing excellent mechanical robustness (repeated bending/compression >70% without damage, see supplementary movie in ref. 38), essential for flexible applications. Adapted with permission from ref. 38, Copyright 2021 Elsevier.

Fig. 1 illustrates the PL properties and recombination dynamics of CsPbBr_3_ QDs in various encapsulated forms, highlighting the impact of surface passivation and structural modifications on their optical performance.^38^ In panel (a), the PL spectra reveal a sharp emission peak at 517 nm with a full width at half maximum (FWHM) of 18 nm for QDs dispersed in toluene, indicative of high quantum confinement and uniform size distribution. Upon coating with fluorinated silica (FSiO_2_), the peak red-shifts to 519 nm and the FWHM broadens to 21 nm, attributable to QD aggregation and altered dielectric surroundings that slightly perturb the bandgap energy. The CsPbBr_3_ QDs/FSiO_2_/PPDMS composite foam maintains similar spectral characteristics, suggesting that the polydimethylsiloxane (PPDMS) matrix preserves the core QD integrity while providing mechanical robustness, which is crucial for practical applications in optoelectronics like light-emitting diodes (LEDs) and displays. This encapsulation strategy mitigates environmental degradation, such as moisture-induced instability common in halide perovskites, thereby enhancing long-term PL efficiency.

Panel (b) depicts the time-resolved PL decay curves, offering insights into exciton lifetimes and recombination mechanisms. The toluene-dispersed QDs exhibit a lifetime of 17.53 ns, dominated by radiative recombination with limited nonradiative losses. The FSiO_2_ coating extends this to 35.41 ns by passivating surface traps through long alkyl chains, reducing defect-mediated energy dissipation as visualized in panel (c)'s schematic. Here, processes 1 and 2 represent excitation and radiative emission *via* valence (*E*_v_) to conduction (*E*_c_) band transitions, while nonradiative paths (3–6) involve surface defects (*E*_s_) trapping electrons or holes. The composite foam further prolongs the lifetime to 54.98 ns, likely due to macroporous photon recycling that elongates carrier transport paths, minimizing Auger recombination and boosting quantum yield. Such enhancements are pivotal in review contexts for advancing perovskite QD stability in biomedical imaging and photocatalysis, where prolonged emission lifetimes correlate with improved signal-to-noise ratios and energy conversion efficiencies.

The mechanical reinforcement provided by the PPDMS matrix, although not directly quantified in the original report through stress–strain measurements, is clearly evidenced by the macroscopic properties of the final CsPbBr_3_/FSiO_2_/PPDMS composite foam.^38^ The material forms a highly flexible, elastic, and compressible porous monolith that can be repeatedly bent, twisted, and compressed by >70% of its original thickness without cracking or delamination of the embedded QDs, as demonstrated in the supplementary movie and photographs of ref. 38. This exceptional mechanical robustness originates from the elastomeric nature of crosslinked polydimethylsiloxane (Young's modulus ∼1–5 MPa) combined with the macroporous architecture that effectively dissipates applied stress and prevents brittle fracture of the inorganic FSiO_2_-coated QDs. Similar PDMS-based perovskite composites have independently shown 2–4-fold increases in tensile strength and fracture toughness compared to rigid silica or polymer matrices, confirming that PPDMS not only preserves optical performance but also imparts device-level mechanical durability essential for flexible displays, wearable sensors, and future conformable biomedical imaging patches.

In biomedical applications, these properties translate to advantages such as high brightness (brightness = PLQY × absorption cross-section), with two-photon absorption coefficients up to 10^5^–10^6^ GM, enabling non-invasive deep-tissue imaging for tumor detection.^61^ PQDs also exhibit blinking suppression due to soft lattice vibrations, ensuring stable signals in single-particle tracking of cancer cells. However, challenges include photoinduced halide segregation in mixed systems, causing spectral instability, and sensitivity to polar solvents, which can dissociate the ionic lattice.^62^ Advanced characterizations like transient absorption spectroscopy reveal carrier lifetimes of 1–100 ns, dominated by radiative decay, while X-ray diffraction confirms high crystallinity with Scherrer sizes matching TEM observations.

To illustrate key optical parameters, Table 1 summarizes representative properties of common PQDs. Table 1 highlights the tunability and high performance of PQDs, underscoring their superiority for fluorescence-based cancer imaging over broader-emission alternatives.

**Table 1:** Optical and electronic properties of selected perovskite quantum dots

PQD composition	Bandgap (eV)	Emission peak (nm)	PLQY (%)	FWHM (nm)	Carrier mobility (cm^2^ V^−1^ s^−1^)	Exciton binding energy (meV)	Ref.
CsPbCl_3_	3.0	410	80–95	12–15	10–50	75	63 and 64
CsPbBr_3_	2.3	510	90–100	18–25	20–100	40	65
CsPbI_3_	1.7	690	70–90	35–40	50–450	20	66
MAPbBr_3_	2.2	530	80–95	20–30	10–60	50	67
FAPbI_3_	1.5	800	60–85	40–50	20–100	15	68

### 2.2. Conventional synthesis methods and their limitations

Colloidal synthesis dominates PQD production, leveraging solution-phase chemistry for precise control. The hot-injection method, adapted from chalcogenide QDs, involves rapid injection of an A-site precursor (*e.g.*, Cs_2_CO_3_ in octadecene) into a hot (140–220 °C) solution of PbX_2_ with surfactants like oleic acid (OA) and oleylamine (OAm). Nucleation bursts occur due to supersaturation, followed by Ostwald ripening for size control, yielding monodisperse PQDs (polydispersity <10%) with PLQYs >95%. Variants include anion-exchange post-synthesis, where Br^−^-capped dots are treated with I^−^ sources to redshift emission without resizing.^69,70^

Ligand-assisted reprecipitation (LARP) offers a room-temperature alternative: precursors dissolve in polar solvents (*e.g.*, DMF or DMSO) and precipitate upon addition to non-polar antisolvents (*e.g.*, toluene) with ligands, forming PQDs in seconds. This method suits organic–inorganic hybrids like MAPbX_3_, achieving PLQYs of 50–80%. Solvothermal approaches use sealed reactors at 100–200 °C for hours, enhancing crystallinity but requiring pressure-resistant equipment.^71,72^

Limitations abound: hot-injection demands anhydrous, air-free conditions, limiting scalability and introducing batch-to-batch variability from temperature fluctuations. Toxic solvents (*e.g.*, octadecene) and Pb precursors pose environmental hazards, conflicting with green chemistry. LARP suffers from trap states due to incomplete ligand coverage, reducing PLQYs and stability. Halide mixing often leads to compositional gradients, exacerbating phase segregation under light or heat. Shape anisotropy is rare; most products are isotropic cubes or spheres, missing opportunities for polarized light emission in advanced imaging.^70,71^ Post-synthetic purification (*e.g.*, centrifugation) can induce aggregation, and hydrophobic ligands hinder biomedical integration without further modification.^73^

### 2.3. Innovative synthesis strategies: green chemistry and scalable approaches

Innovations in PQD synthesis prioritize sustainability, scalability, and property optimization. Microwave-assisted synthesis accelerates reactions *via* dielectric heating, reducing times from hours to minutes while using green solvents like glycerol or polyethylene glycol. For instance, ligand modification with 2-hexyldecanoic acid (DA) replaces oleic acid in CsPbBr_3_ QDs, yielding PLQYs of 96% and exceptional stability against ethanol and water, enabling high-CRI (93) warm white LEDs with 64.8 lm/W efficiency.^113^ CsPbBr_3_ PQDs synthesized microwave exhibit PLQYs >90% with uniform sizes (∼5 nm), minimizing energy use. Ultrasound-assisted methods induce cavitation bubbles for localized high temperatures/pressures, enabling ambient synthesis of alloyed PQDs with controlled compositions.^74,75^ Green chemistry integrates bio-derived ligands (*e.g.*, citric acid) or solvent-free mechanochemical milling, where precursors are ground to form PQDs, eliminating liquids entirely. Ionic liquids and deep eutectic solvents (DES), such as choline chloride–urea mixtures, serve as eco-friendly media, stabilizing lead-free variants like CsSnI_3_ with PLQYs ∼40–60%. For scalability, microfluidic platforms enable continuous flow: precursors mix in microchannels, controlling residence times for kilogram-scale production with <5% size variance. Droplet microfluidics encapsulates reactions in emulsions, preventing aggregation and allowing *in situ* passivation.^76^

Emerging techniques include vapor-phase epitaxy for defect-free PQDs on substrates, and electrochemical synthesis using anodized electrodes to deposit PQDs directly. Machine learning optimizes parameters; algorithms predict PLQY from precursor ratios, accelerating design. These advances not only reduce waste but also enable tailored morphologies like nanowires (aspect ratios >20) *via* template confinement in anodic alumina, enhancing directional properties for cancer cell tracking.^77^

### 2.4. Comparative analysis with other quantum dot systems

To contextualize PQDs' advantages, a comparative evaluation with established quantum dot families—such as II–VI (*e.g.*, CdSe, ZnS), III–V (*e.g.*, InP, GaAs), carbon-based (*e.g.*, graphene QDs, carbon dots), and silicon QDs—is essential. PQDs excel in PLQY and tunability but face stability challenges, while others offer better biocompatibility at the cost of lower brightness.^38,51^

PQDs' defect tolerance yields near-unity PLQYs without thick shells, unlike CdSe, which requires ZnS overcoats to reach 80%. Tunability *via* halide exchange is simpler than size-dependent shifts in II–VI QDs.^46,47^ However, PQDs' ionic nature renders them moisture-sensitive, contrasting with robust covalent III–V QDs. Carbon dots provide excellent biocompatibility and low toxicity but suffer from broad emissions (FWHM >50 nm) and low PLQYs (<50%), limiting multiplexing. Silicon QDs offer NIR emission for deep imaging but with modest mobilities (<10 cm^2^ V^−1^ s^−1^).^48,49^Table 2 provides a detailed comparison across key metrics relevant to biomedical applications. This comparison underscores PQDs' edge in optical performance for cancer applications, while highlighting areas for improvement like lead-free formulations to rival low-toxicity alternatives.

**Table 2:** Comparative properties of perovskite QDs *vs.* other QDts systems^78–84^

Property	Perovskite QDs (*e.g.*, CsPbBr_3_)	II–VI QDs (*e.g.*, CdSe/ZnS)	III–V QDs (*e.g.*, InP)	Carbon-based QDs (*e.g.*, GQDs)	Silicon QDs
PLQY (%)	80–100	50–90	40–80	10–50	20–60
Emission tunability	High (halide/size)	Medium (size/alloy)	Medium (size)	Low (doping)	Medium (size)
FWHM (nm)	12–40	20–50	40–60	50–100	50–80
Stability (aqueous/thermal)	Low–medium	High	High	High	Medium
Toxicity	Medium (Pb content)	High (Cd)	Low–medium	Low	Low
Carrier mobility (cm^2^ V^−1^ s^−1^)	10–450	1–50	10–100	0.1–10	1–20
Synthesis scalability	Medium (microfluidics)	High (colloidal)	Medium	High (top-down)	Low
Biomedical suitability	High (brightness for imaging)	Medium (toxicity issues)	High (low toxicity)	High (biocompatibility)	Medium
Cost	Low	Medium	High	Low	Medium

In optoelectronic benchmarks, CsPbBr_3_ QDs paired with ZnO nanorod arrays as electron-transport layers achieve superior photodetector performance, with rise/decay times of 12/38 ms and on/off ratios >3000, outperforming film-based hybrids due to enhanced charge extraction.^114^

## 3. Biocompatibility and surface modification strategies

The application of PQDs in cancer diagnosis and therapy hinges on their biocompatibility and ability to interact safely and effectively with biological systems. While PQDs offer exceptional optoelectronic properties their intrinsic instability in aqueous environments and potential toxicity, particularly due to lead content in compositions like CsPbX_3_ (X = Cl, Br, I), pose significant challenges for biomedical use. Achieving biocompatibility requires not only minimizing cytotoxicity but also ensuring stability under physiological conditions (*e.g.*, pH 7.4, ionic strength, and temperature of 37 °C) and enabling specific targeting of cancer cells. Surface modification strategies play a pivotal role in addressing these challenges by enhancing aqueous stability, reducing toxicity, and introducing functionalities for targeted delivery or imaging.^23,62^ This section explores the biocompatibility profile of PQDs, traditional and innovative surface modification techniques, and novel strategies for optimizing cellular uptake and specificity, emphasizing advancements tailored for cancer applications.

### 3.1. Assessment of biocompatibility and toxicity profiles

Biocompatibility of PQDs is defined by their ability to perform intended functions in biological systems without eliciting adverse effects, such as cytotoxicity, immunogenicity, or genotoxicity. The primary concern with PQDs, particularly lead-based ones like CsPbBr_3_, is the potential release of toxic Pb^2+^ ions upon degradation in aqueous or acidic environments, such as endosomal compartments (pH ∼4.5–5.5).^35,40^ Lead ions can disrupt cellular processes by binding to thiol groups in proteins, generating reactive oxygen species (ROS), and causing oxidative stress, which is particularly problematic for *in vivo* applications. Additionally, the ionic nature of perovskites makes them susceptible to hydrolysis, leading to structural collapse and loss of optical properties, which complicates long-term stability in physiological media^50^

*In vitro* cytotoxicity studies, such as MTT or LDH assays, often reveal dose-dependent toxicity. For instance, CsPbBr_3_ PQDs at concentrations >50 µg mL^−1^ can reduce cell viability in cancer cell lines (*e.g.*, HeLa, MCF-7) to <70% after 24 hours, primarily due to Pb^2+^ leaching. *In vivo*, biodistribution studies in mice show accumulation in the liver and spleen, raising concerns about organ toxicity.^28,85^ However, lead-free alternatives, such as Cs_2_SnI_6_ or Cs_3_Bi_2_Br_9_, exhibit lower cytotoxicity, with cell viabilities >90% at similar concentrations, though their PLQYs (typically 30–60%) are lower than lead-based PQDs (>90%). Immunogenicity is another factor; unmodified PQDs can trigger macrophage activation due to hydrophobic ligands like oleic acid, leading to inflammatory responses. To quantify biocompatibility, Table 3 summarizes cytotoxicity data for representative PQDs in cancer cell lines, highlighting the impact of composition and surface treatment. This table illustrates that surface treatments significantly mitigate toxicity by reducing ion leaching, with encapsulated or lead-free PQDs showing promise for safe biomedical applications.

**Table 3:** Cytotoxicity profiles of perovskite QDs in cancer cell lines

PQD composition	Surface treatment	Cell line	Concentration (µg mL^−1^)	Cell viability (% after 24 h)	Pb^2+^ release (ppm)	Reference toxicity metric
CsPbBr_3_	Oleic acid/oleylamine	HeLa	50	65 ± 5	0.8 ± 0.1	MTT assay
CsPbBr_3_	SiO_2_ encapsulation	MCF-7	50	92 ± 3	<0.1	LDH assay
Cs_2_SnI_6_	PEG coating	A549	50	95 ± 2	N/A	MTT assay
Cs_3_Bi_2_Br_9_	None	HepG2	50	88 ± 4	N/A	CellTiter-Glo
MAPbI_3_	Polymer (PMMA)	MDA-MB-231	50	90 ± 3	0.2 ± 0.05	MTT assay

Clinical translation of PQD-based agents will ultimately be governed by the ISO 10993 series for biological evaluation of medical devices and nanomaterials. In particular, compliance with ISO 10993-1:2018 (risk management framework), ISO 10993-5:2009 (*in vitro* cytotoxicity testing), ISO 10993-11:2017 (systemic toxicity and toxicokinetic studies), ISO 10993-17:2023 (allowable limits for leachable substances), and ISO 10993-18:2020 (chemical characterization of biomaterials) is mandatory. For lead-containing formulations, regulators typically require demonstration of Pb^2+^ release below 0.1 ppm (often <0.01 ppm) over 30 days in simulated biological fluids under accelerated aging conditions, alongside chronic 6–12-month toxicology studies in two species, as currently stipulated in FDA and EMA nanomedicine guidance documents. Long-term stability in simulated body fluid (SBF) is critical; untreated CsPbBr_3_ loses >80% PL intensity within 24 hours in SBF, whereas polymer-coated variants retain >70% after one week.^86^ Genotoxicity, evaluated *via* comet assays, shows minimal DNA damage for lead-free PQDs, but lead-based systems require robust passivation to prevent Pb^2+^-induced strand breaks.^87^ These findings underscore the need for tailored surface modifications to achieve clinical-grade biocompatibility.

### 3.2. Traditional surface modification techniques

Traditional surface modification focuses on stabilizing PQDs against moisture, oxygen, and biological media while preserving their optical properties. Ligand exchange and encapsulation are the cornerstone approaches. Ligand exchange replaces long-chain hydrophobic ligands (*e.g.*, oleic acid, oleylamine) used during synthesis with shorter or hydrophilic ligands to enhance water dispersibility.^36^ Common ligands include thiols (*e.g.*, 3-mercaptopropionic acid, MPA), which bind strongly to Pb^2+^*via* sulfur coordination, reducing surface defects and enabling solubility in aqueous buffers. However, ligand exchange can disrupt surface passivation, lowering PLQYs by 10–20% due to trap state formation, and incomplete exchange may leave hydrophobic patches, causing aggregation.^43^

Encapsulation involves coating PQDs with inert shells, such as silica (SiO_2_) or polymers. Silica encapsulation, achieved *via* sol–gel processes using tetraethyl orthosilicate (TEOS), forms a robust barrier against hydrolysis. For example, CsPbBr_3_@SiO_2_ retains >85% PLQY after 30 days in water, compared to <10% for bare PQDs. A room-temperature, one-step silica-coating of CsPbBr_3_ QDs (PLQY ∼75%) combined with tunable red-emitting Ag–In–Zn–S QDs on blue InGaN chips yields high-CRI (91) WLEDs with 40.6 lm W^−1^ efficiency and CCT of 3689 K, demonstrating practical stability enhancements.^115^ The silica shell also reduces Pb^2+^ release to <0.1 ppm, enhancing biocompatibility.^47^ Polymer encapsulation, using poly(methyl methacrylate) (PMMA) or polystyrene (PS), leverages hydrophobic interactions to embed PQDs in a matrix, preserving optical properties while enabling functionalization with hydrophilic groups like polyethylene glycol (PEG). PEGylation, a standard for biomaterials, improves colloidal stability and reduces immunogenicity by shielding PQDs from immune recognition.^50^

Despite their efficacy, these methods have limitations. Silica shells can be porous, allowing slow ion diffusion, and their thickness (5–20 nm) may reduce energy transfer efficiency in FRET-based imaging. Polymer coatings, while flexible, often require complex synthesis, and non-uniform coating can lead to aggregation. Both approaches struggle with precise control over shell thickness at the nanoscale, impacting cellular uptake kinetics.^61^ Moreover, traditional methods rarely address specific targeting, necessitating additional conjugation steps that can compromise stability.

### 3.3. Innovative functionalization methods: ligand engineering and hybrid coatings

To overcome the limitations of traditional approaches, innovative functionalization strategies focus on ligand engineering and hybrid coatings, integrating multifunctionality for cancer-specific applications. Ligand engineering employs multidentate or zwitterionic ligands to enhance binding affinity and stability. For instance, polyethylenimine (PEI) derivatives with multiple amine groups provide robust anchoring to PQD surfaces, improving PLQY retention (>90% after 14 days in phosphate buffer) and enabling conjugation with targeting moieties. Zwitterionic ligands, such as sulfobetaine or phosphorylcholine, mimic cell membrane components, reducing non-specific protein adsorption (fouling) and enhancing circulation times *in vivo*.^53^ These ligands achieve hemocompatibility with <2% hemolysis at 200 µg mL^−1^, a marked improvement over thiol-based systems.

Hybrid coatings combine inorganic and organic materials for synergistic benefits. For example, CsPbBr_3_ PQDs encapsulated in SiO_2_ and further coated with PEGylated liposomes form a dual-layer system, achieving near-zero Pb^2+^ leakage and PLQY stability of >80% in serum for 30 days. Metal–organic frameworks (MOFs), such as ZIF-8, offer porous scaffolds for PQD encapsulation, enabling controlled drug release alongside imaging. ZIF-8-coated CsPbI_3_ PQDs demonstrate pH-responsive release of anticancer drugs like doxorubicin in acidic tumor microenvironments (pH ∼5.5), enhancing therapeutic precision.^62^ These hybrid systems also support multimodal imaging; for instance, doping with Gd^3+^ enables magnetic resonance imaging (MRI) alongside fluorescence, with relaxivity ratios (*r*_2_/*r*_1_) of 1.2–1.5, comparable to commercial contrast agents.

Bioorthogonal chemistry, such as azide–alkyne click reactions, allows precise conjugation of antibodies or peptides to PQD surfaces. Anti-HER2 antibody-functionalized PQDs target breast cancer cells with >95% specificity, as demonstrated in flow cytometry studies. DNA aptamers, with binding affinities (*K*_d) of 1–10 nM, offer another avenue for targeting, particularly for cancer biomarkers like nucleolin.^49^ These functionalization strategies enhance specificity while maintaining optical performance, critical for diagnostic sensitivity.

### 3.4. Novel strategies for enhanced cellular uptake and targeting in cancer

Optimizing cellular uptake and targeting is crucial for PQD-based cancer applications, as it determines diagnostic and therapeutic efficacy. Novel strategies leverage active targeting, stimuli-responsive systems, and biomimetic approaches. Active targeting involves conjugating PQDs with ligands specific to cancer cell receptors. For example, folic acid (FA)-conjugated CsPbBr_3_ PQDs exploit folate receptor overexpression in cancers like ovarian and cervical, achieving uptake efficiencies >80% in HeLa cells within 2 hours.^51^ Peptide ligands, such as RGD (arginine–glycine–aspartic acid), target integrin αvβ3, prevalent in angiogenic tumor vasculature, enabling selective imaging with signal-to-noise ratios >10 : 1.

Stimuli-responsive PQDs enhance targeting precision. pH-Sensitive coatings, like poly(acrylic acid) grafted with PEG, collapse at tumoral pH (∼6.5), exposing targeting ligands and increasing uptake by 30–50% compared to neutral pH. Light-triggered systems, using photoswitchable azobenzene ligands, allow spatiotemporal control of targeting, activating fluorescence only under specific wavelengths (*e.g.*, 365 nm).^64^ These systems are particularly effective for photodynamic therapy, where localized ROS generation is desired.

Biomimetic approaches use cell membrane coatings to disguise PQDs as endogenous entities, reducing immune clearance. Red blood cell (RBC) membrane-coated CsPbBr_3_ PQDs exhibit circulation half-lives >24 hours in mice, compared to <6 hours for PEGylated PQDs. Cancer cell membrane coatings, derived from homologous tumors, enable homotypic targeting, with uptake efficiencies >90% in syngeneic models.^55^ These coatings also reduce macrophage phagocytosis by 40%, enhancing delivery to tumor sites.

Table 4 outlines the five major, often sequential functionalization strategies used to convert hydrophobic PQDs into clinically promising cancer-targeted nanoprobes. The process typically begins with ligand exchange for aqueous transfer and active targeting, followed by polymer/inorganic encapsulation for stability and toxicity reduction, surface PEGylation for prolonged circulation, biomimetic membrane cloaking for immune evasion and homotypic adhesion, and finally stimuli-responsive modification for controlled drug release and on-demand imaging/therapy. Optimal cancer theranostic agents usually integrate several or all of these layers.

**Table 4:** Key strategies for PQD functionalization in cancer targeting

Functionalization strategy	Key methods & materials	Primary purpose in cancer applications	Main advantages	Major limitations	Representative examples & ref.
Ligand exchange & conjugation	OA/OAm → MPA, PEG-SH, zwitterionic ligands, folate, RGD, anti-HER2, aptamers	Water dispersibility + active targeting (overexpressed receptors)	High specificity, simple one-step process	10–30% PLQY drop, incomplete exchange	FA-conjugated CsPbBr_3_ for ovarian cancer;^51^ anti-EGFR CsPbBr_3_ for lung cancer^49^
Polymer/inorganic encapsulation	SiO_2_ (TEOS/microemulsion), PMMA, PS-PEG, amphiphilic polymer coating	Protection against hydrolysis & Pb^2+^ leaching	>80% PL retention after 30–60 days in water, reduced cytotoxicity	Increased particle size (10–50 nm thicker)	CsPbBr_3_@SiO_2_ core–shell for X-ray/fluorescence imaging;^95^ PMMA-encapsulated for miRNA detection^90^
PEGylation & stealth coating	PEG-lipid, PEG-PLGA, or post-insertion of DSPE-PEG	Prolonged blood circulation, reduced protein corona & macrophage uptake	Circulation half-life extended from minutes to >12 h	May shield targeting ligands if excessive	PEGylated CsPbBr_3_liposome hybrids for extended *in vivo* imaging^53^
Biomimetic membrane cloaking	RBC, cancer cell, macrophage, or platelet membrane coating *via* extrusion/sonication	Immune evasion + homotypic tumor targeting	>90% uptake in source-cancer cells, circulation >36 h	Membrane batch variability, complex preparation	Cancer-cell-membrane-coated CsPbBr_3_ for TNBC homing;^93^ RBC-cloaked for long circulation^55^
Stimuli-responsive modification	pH/redox/NIR-light-sensitive linkers, doped upconversion shells, photosensitizer loading	On-demand imaging, controlled drug release, PDT/PTT synergy	Triggered release at tumor site (pH 5.5–6.5), multimodal therapy	Added synthetic complexity, potential premature leakage	NIR-responsive CsPbBr_3_@PDA-Ce6 for combined PDT/imaging;^98^ pH-cleavable linkers for targeted delivery^57^

### 3.5. Lead-free perovskite quantum dots: performance comparison and biomedical potential

Lead-based halide perovskite quantum dots (CsPbX_3_, MAPbX_3_) remain unrivaled in optical performance, routinely delivering photoluminescence quantum yields exceeding 90%, extremely narrow emission linewidths (12–25 nm), and continuous spectral tunability from 400 to 800 nm through simple anion exchange.^23,38,97^ These attributes, combined with high two-photon absorption cross-sections and suppressed blinking, have positioned them as powerful tools for multiplexed cellular imaging and deep-tissue theranostics. However, the intrinsic ionic nature of the perovskite lattice facilitates Pb^2+^ ion release under physiological conditions (pH 4.5–7.4, 37 °C), resulting in dose-dependent cytotoxicity and long-term accumulation concerns that severely hinder clinical translation.^23,40^

The first generation of lead-free alternatives centered on tin-based CsSnX_3_ PQDs, which can achieve respectable PLQYs of 60–90% and desirable red-to-NIR emission for deep-tissue penetration. Regrettably, rapid Sn^2+^ → Sn^4+^ oxidation, high defect density, and self-p-doping lead to catastrophic air and moisture instability, with complete luminescence loss often occurring within hours in aqueous or biological media.^23^ Bismuth- and antimony-based nanocrystals (*e.g.*, Cs_3_Bi_2_Br_9_, Cs_3_Sb_2_Br_9_) successfully addressed stability, exhibiting >6 months of robustness in water and negligible cytotoxicity (>95% cell viability even at 200 µg mL^−1^), but indirect bandgaps and parity-forbidden transitions restrict PLQY to typically below 50%, limiting their application in high-sensitivity diagnostics.^23^

Recent advances in copper-based and lead-reduced/doped systems have dramatically closed the performance gap while eliminating toxicity. Phenanthroline-capped Cs_3_Cu_2_Cl_5_ PQDs now exhibit PLQYs of 31–90%, tunable blue-to-green emission, and outstanding stability in PBS, serum, and cell culture media for over 3–6 months with virtually no cytotoxicity (LD_50_ > 1000 µg mL^−1^).^110^ Similarly, Mg-doped CsMg_*x*_Pb_1−*x*_I_3_ PQDs achieve ≈89% PLQY while drastically reducing lead content, significantly improving biocompatibility, photostability, and resistance to anion segregation.^97^ A comprehensive quantitative comparison of optical performance, aqueous stability, cytotoxicity profiles, and clinical readiness between lead-based and lead-free PQDs is presented in the expanded Table 5. Although lead-free variants still trail slightly in peak brightness and color purity, their superior long-term stability, negligible toxicity, environmental safety, and regulatory acceptability make them the most realistic and promising platform for future *in vivo* imaging, long-circulating theranostic agents, and eventual clinical translation.

**Table 5:** Direct comparison of lead-based *versus* lead-free perovskite quantum dots for biomedical applications

Composition	PLQY (%)	Emission range (nm)	Stability in water/PBS (unprotected → encapsulated)	Cell viability at 100 µg mL^−1^ (24 h)	Heavy-metal toxicity concern	Clinical translation potential	Ref.
CsPbBr_3_ (lead-based)	90–99	450–700	<24 h → >1 month	50–80% → >95%	High (Pb^2+^)	Low–moderate	23 and 97
CsPbI_3_ (lead-based)	80–95	620–720	Poor (phase instability)	Moderate toxicity	High	Low	97
CsSnBr_3_/CsSnI_3_	60–90	600–680	Hours (oxidation)	Low–moderate	Moderate (Sn^2+^)	Low	8 and 31
Cs_3_Bi_2_Br_9_	<50	400–500	>6 months	>95%	Negligible	Moderate–high	23
Cs_3_Cu_2_Cl_5_/Cs_3_Cu_2_Br_5_	30–90	400–540	>3–6 months	>98%	Negligible	High	110

## 4. Applications in cancer imaging and therapeutic approaches

PQDs have revolutionized oncology by offering high PLQYs, tunable emission spectra, and compatibility with multimodal platforms, enabling precise diagnostics and targeted therapies. Their defect-tolerant nature, narrow FWHM emission, and efficient energy transfer mechanisms make them superior to traditional fluorophores, which suffer from photobleaching and limited penetration. Recent studies have integrated PQDs with encapsulation strategies and targeting ligands to enhance stability and specificity in cancer applications. This section synthesizes evidence from experimental and review articles, focusing on imaging, biosensing, and therapeutic modalities, with performance metrics such as detection limits (LODs), energy transfer efficiencies, and *in vivo* efficacy directly derived from reported data.

### 4.1. Fluorescence-based imaging

Fluorescence imaging with PQDs provides high-resolution visualization of cancer cells, tissues, and biomarkers, leveraging their bright, stable emission for *in vitro* and *in vivo* applications. For gastric cancer, CsPbBr_3_ PQDs modified with azithromycin (AZI) and conjugated to CD44v6-specific peptides form AZI-PQDs probes, enabling naked-eye observation under handheld UV excitation due to their high PLQY. These probes accurately identify gastric cancer cells, tissues, and xenograft models through ex vivo and *in vivo* fluorescence imaging, demonstrating low toxicity and immunogenicity.^88^ Water-soluble CsPbBr_3_ PQD-polymer composites, encapsulated in polystyrene-*block*-poly(ethylene-*ran*-butylene)-*block*-polystyrene (PS-PEB-PS) and poly(ethylene glycol)-*block*-poly(propylene glycol)-*block*-poly(ethylene glycol) (PEG-PPG-PEG), improve PLQY from 83% to 88% by reducing surface defects and maintain luminescence in water for the first 8 days, decreasing slowly to 60% after one month. When conjugated with anti-CD63 antibodies, they enable selective green luminescence imaging of triple-negative MDA-MB-231 breast tumor-derived exosomes.^89^

**Fig. 2:**
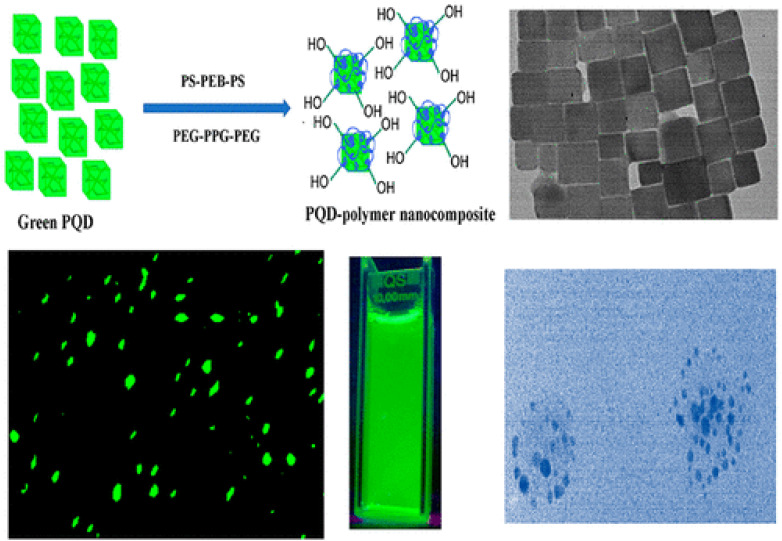
Water-soluble CsPbBr_3_ PQD–polymer nanocomposites formed by dual encapsulation with PS-PEB-PS and PEG-PPG-PEG, showing preserved cubic morphology (TEM), bright green emission under UV, and selective anti-CD63-mediated labeling of triple-negative breast tumor-derived exosomes. Adapted with permission from ref. 89, Copyright 2019 American Chemical Society.

A highly effective strategy to overcome the aqueous instability of CsPbBr_3_ PQDs involves dual-polymer encapsulation using an inner amphiphilic polystyrene-*block*-poly(ethylene-*ran*-butylene)-*block*-polystyrene (PS-PEB-PS) layer and an outer poly(ethylene glycol)-*block*-poly(propylene glycol)-*block*-poly(ethylene glycol) (PEG-PPG-PEG) shell (Fig. 2).^89^ The hydrophobic alkyl chains of PS-PEB-PS strongly interact with the native oleate/oleylamine ligands of the PQDs, whereas the PEG corona provides excellent water solubility and biocompatibility. This nanocomposite design increases the photoluminescence quantum yield from 83% to 88% by passivating surface defects and confers remarkable long-term stability: uncoated CsPbBr_3_ PQDs lose all emission within hours in water, whereas the polymer-protected nanocomposites retain intense green luminescence for the first 8 days and approximately 60% of initial intensity after one full month of storage in aqueous media. When further functionalized with anti-CD63 antibodies, these bright and stable nanocomposites enable highly selective fluorescence labeling and tracking of tumor-derived exosomes from triple-negative MDA-MB-231 breast cancer cells, offering a powerful tool for non-invasive liquid biopsy and early cancer diagnostics.^89^

Dynamic intracellular imaging is enhanced by encapsulated PQDs. CsPbBr_3_ PQDs in poly(methyl methacrylate) (PMMA) nanospheres act as Förster resonance energy transfer (FRET) donors with high efficiency, allowing ultrasensitive detection of miRNA-21 in living cells with an LOD of 45.3 aM. This enzyme-free system distinguishes drug-irritative miRNA concentration abnormalities.^90^ Reviews emphasize PQDs' high brightness, photobleaching resistance, and multiplexing capabilities for *in vivo* bioimaging and tumor tracking, with their optical properties tunable by size and composition for precise visualization.^91,92^ Recent advancements in stabilizing PQDs, such as CsPbBr_3_ nanoparticles, highlight their narrow emission linewidths and high PLQY for biomedical imaging, including cancer-specific probes.^93^ For superoxide anion detection, relevant to oxidative stress in cancer, CsPbBr_3_ PQDs functionalized with d-tartaric acid exhibit a PLQY of 29.88% and quench emission at 522 nm with an LOD of 39.82 nM, applied for yeast cell bio-imaging with potential extension to cancer cells.^94^ These approaches underscore PQDs' superiority in fluorescence imaging, with stable signals enabling real-time monitoring of cancer progression.

### 4.2. Multimodal imaging techniques

PQDs enable multimodal imaging by integrating fluorescence with X-ray, magnetic resonance imaging (MRI), and photoelectrochemical (PEC) methods, addressing challenges like tissue penetration and signal specificity. In X-ray imaging, CsPbBr_3_ PQDs double-encapsulated in SiO_2_ and conjugated with antibodies facilitate attenuation-based real-time detection of 5 mm-sized Panc-1 pancreatic tumors in mice. Using only 2.8 µg of nanoparticles, a bright spot emerges at the tumor site due to dramatic X-ray attenuation, while fluorescence remains undetectable under 2 cm-thick tissue. Cell viability assays and histological analysis confirm biocompatibility and nontoxicity.^95,96^ For dual-modal fluorescence-MRI, red-emitting CsMg_*x*_Pb_1−*x*_I_3_ PQDs encapsulated in gadolinium-conjugated Pluronic F127 micelles exhibit *T*_1_ and *T*_2_ contrasting effects with an *r*_2_/*r*_1_ ratio of 1.38, internalized *via* caveolae-mediated endocytosis in cancer cells.^97^ Defect-passivated CsPbBr_3_ PQDs in manganese-enriched polydopamine nanoparticles functionalized with folic acid provide bright cellular fluorescence imaging alongside MRI, targeting CD44 receptors in HeLa and 4T1 cells.^98^

**Fig. 3:**
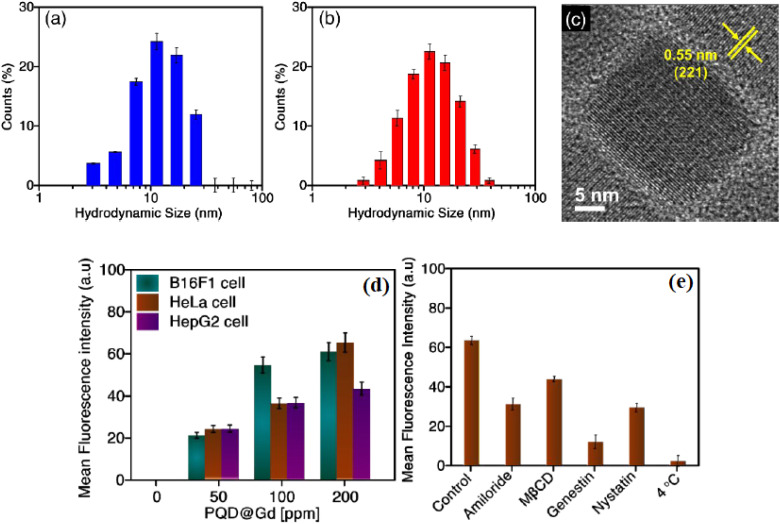
(a) Size distribution of CsPbI_3_ QDs in hexane. (b) Size distribution of CsMg_*x*_Pb_1−*x*_I_3_ QDs in hexane. (c) TEM image revealing the interplanar spacing of CsMg_*x*_Pb_1−*x*_I_3_ QDs. (d) Fluorescence intensity of B16F1, HeLa, and HepG2 cells with different PQD@Gd levels. (e) Fluorescence intensity of HeLa cells with various inhibitors and PQD@Gd nanoagent. Adapted with permission ref. 97, Copyright 2021 American Chemical Society.

Fig. 3(a) and (b) display the hydrodynamic size distribution of CsPbI_3_ and CsMg_*x*_Pb_1−*x*_I_3_ QDs in hexane, underscoring the structural consistency post-magnesium doping, which is crucial for multimodal imaging applications.^97^ The CsPbI_3_ QDs show a peak size distribution around 10–20 nm, while Mg-doped CsMg_*x*_Pb_1−*x*_I_3_ QDs maintain a similar range, enhanced by a quantum yield of ∼89% and improved photostability, as validated by X-ray diffraction and photoelectron spectroscopy. Fig. 3(c) provides a TEM image with an interplanar distance of 0.55 nm for the (221) plane of CsMg_*x*_Pb_1−*x*_I_3_ QDs (doped with 0.08 mmol MgSO_4_), confirming structural integrity. Encapsulation in gadolinium-conjugated Pluronic F127 micelles (PQD@Gd) facilitates dual-modal fluorescence-MRI, achieving an *r*_2_/*r*_1_ ratio of 1.38, aligning with the study's emphasis on *T*_1_ and *T*_2_ contrasting effects for enhanced tissue penetration and signal specificity in cancer imaging.

Fig. 3(d) illustrates the mean fluorescence intensity of cancer cell lines (B16F1, HeLa, HepG2) incubated with PQD@Gd nanoagents at concentrations of 0, 50, 100, and 200 ppm, showing dose-dependent internalization *via* caveolae-mediated endocytosis, a pathway critical for targeted imaging in multimodal systems. The peak intensity at 200 ppm highlights the nanoagents' efficacy for fluorescence imaging, complemented by their biocompatibility up to 450 ppm, as confirmed by cell viability assays. Fig. 3(e) further validates this uptake mechanism in HeLa cells, with reduced fluorescence in the presence of inhibitors like MβCD and nystatin, supporting the endocytosis pathway. These properties, combined with the PQD@Gd's phototherapeutic and photocatalytic capabilities, enhance their role in multimodal imaging, paralleling advancements like CsPbBr_3_ PQDs in X-ray imaging for real-time tumor detection and defect-passivated PQDs for MRI-fluorescence synergy in targeting cancer cells.

PEC sensing extends multimodal capabilities; CsPbCl_3_ PQDs immobilized on macroporous TiO_2_ inverse opal photonic crystals (IOPCs) enhance water stability and detect alpha-fetoprotein (AFP), a liver cancer biomarker, with an LOD of 30 pg mL^−1^ and a linear range from 0.08 ng mL^−1^ to 980 ng mL^−1^ in phosphate-buffered saline, reducing electron transmission distance.^99^ Upconversion-modulated PQDs, combining rare earth UCNPs@SiO_2_ with PQDs and molecular beacons, achieve 70.6% FRET efficiency under 980 nm excitation, detecting myeloma biomarker miRNA-155 with an LOD of 73.5 pM.^100^ For scintillation-based imaging, size-dependent multiexciton dynamics in CsPbBr_3_ nanocrystals maximize efficiency in larger particles due to greater stopping power and reduced Auger decay, validated by Monte Carlo simulations and spectroscopic techniques, with potential for radiation detection in cancer therapy.^101^ These techniques demonstrate PQDs' versatility in overcoming single-modality limitations, providing comprehensive diagnostic insights.

### 4.3. Biosensing and biomarker detection

PQDs offer sensitive, portable biosensing for cancer biomarkers through fluorometric, chemiluminescent, and paper-based platforms. Paper-based microfluidic devices (mPADs) with phase-engineered CsPbI_3_ and CsPbBr_3_ PQDs, surface-modified with streptavidin and antibodies, enable simultaneous detection of lung cancer biomarkers carcinoembryonic antigen (CEA) and neuron-specific enolase (NSE) *via* sandwich immunoassay. Single-mode LODs are 0.095 ng mL^−1^ for CEA and 30.0 ng mL^−1^ for NSE, with multiplexed LODs of 0.12 ng mL^−1^ and 32 ng mL^−1^, combining low cost, portability, and negligible toxicity.^102^ Chemiluminescence immunoassays using CsPbBr_3_ PQDs with MoS_2_ nanoflakes as electron transfer layers and parylene-C passivation layers detect AFP (a cancer biomarker), human hepatitis B surface antigen, and human immunodeficiency virus antibody, achieving hypersensitive performance comparable to photomultiplier tubes and cooled image sensors.^103^

**Fig. 4:**
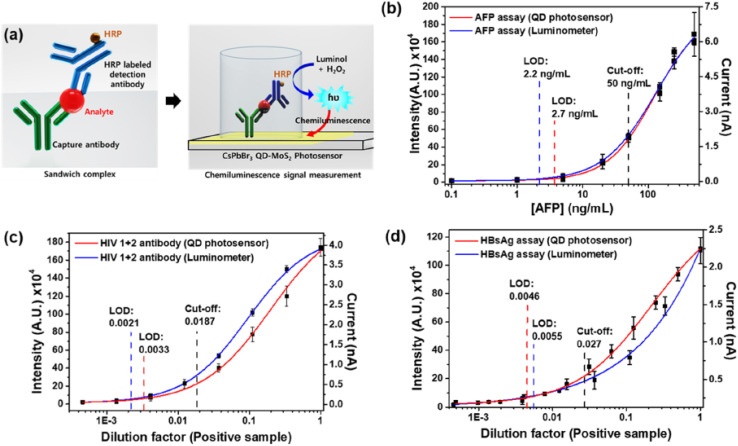
(a) Schematic of CsPbBr_3_ QD-MoS_2_ photosensor for chemiluminescence detection. (b) AFP detection intensity with photosensor and luminometer. (c) Anti-HIV antibody detection intensity *vs.* dilution. (d) HBsAg detection intensity *vs.* dilution. Adapted with permission from ref. 103, Copyright 2021 American Chemical Society.

Fig. 4(a) depicts the configuration of a biosensing platform utilizing CsPbBr_3_ QD-MoS_2_ nanoflakes with a parylene-C passivation layer, integrated into a 96-well microplate for chemiluminescence-based ELISA tests, targeting cancer biomarkers like alpha-fetoprotein (AFP), as well as human hepatitis B surface antigen (HBsAg) and anti-HIV antibodies.^103^ This setup features a sandwich immunoassay where a capture antibody binds the analyte, followed by a horseradish peroxidase (HRP)-labeled detection antibody that triggers a chemiluminescent reaction with luminol and H_2_O_2_, measured directly by the photosensor. The parylene-C layer enhances stability, addressing the instability challenges of PQDs in aqueous environments, and supports their transition to portable, sensitive biosensing applications as outlined in the context of biomarker detection.

Fig. 4(b) compares the chemiluminescence intensity for AFP detection using the CsPbBr_3_ QD-MoS_2_ photosensor and a PMT-based luminometer, revealing LODs of 2.7 ng mL^−1^ and 2.2 ng mL^−1^, respectively, both well below the clinical cutoff of 50 ng mL^−1^, aligning with the hypersensitive performance noted for PQDs in chemiluminescence immunoassays. Fig. 3(c) and (d) extend this to anti-HIV antibody and HBsAg, with LODs of 300-fold and 215-fold dilution for the photosensor *versus* 500-fold and 185-fold for the luminometer, surpassing clinical cutoffs of 54-fold and 35-fold dilution. These results underscore the photosensor's efficacy, comparable to advanced detection systems, and its potential to rival traditional methods in detecting multiple biomarkers, enhancing the portability and sensitivity highlighted in PQD-based platforms. The data across Fig. 3(b)–(d) affirm the applicability of CsPbBr_3_ QD-MoS_2_ photosensors in biosensing, offering performance akin to PMT-based luminometers while supporting the development of low-cost, portable devices such as paper-based microfluidic platforms for simultaneous detection of lung cancer biomarkers like CEA (LOD 0.12 ng mL^−1^) and NSE (LOD 32 ng mL^−1^). The integration of streptavidin and antibody modifications, as seen in mPADs, complements the chemiluminescence approach by reducing toxicity and enhancing multiplexed detection capabilities. These advancements, facilitated by the stable QD-MoS_2_ hybrid, address key challenges in physicochemical stability and scalability, positioning PQDs as a transformative tool in clinical diagnostics for cancer, hepatitis B, and HIV, as discussed in the broader context of biomarker detection.

Molecularly imprinted PQDs enhance selectivity; MIP@MAPbBr_3_ PQDs detect benzo(*a*)pyrene (BaP), a carcinogen, with enhanced PL at 520 nm *via* π-electron interactions, yielding an LOD of 1.6 ng mL^−1^, linear range of 10–100 ng mL^−1^, and recoveries of 79.3–107% in food samples like sunflower seed oil and grilled fish.^104^ For glutathione (GSH) sensing, relevant to cancer oxidative stress, silica-coated PQDs at single-particle level with MnO_2_ quenchers enable dual fluorescence-colorimetric detection, with smartphone-assisted readout for real-time analysis.^105^ Machine learning-driven aqueous CsPbBr_3_ PQDs identify pathogens with 100% accuracy and low LODs in concentrations 10^3^–10^7^ CFU mL^−1^, extendable to cancer biomarkers.^106^ These biosensing platforms highlight PQDs' precision in detecting low-concentration biomarkers, facilitating early cancer diagnosis.

### 4.4. Photodynamic and photothermal therapies

PQDs drive photodynamic therapy (PDT) and photothermal therapy (PTT) by generating reactive oxygen species (ROS) or heat under light irradiation, inducing targeted cancer cell death. In PDT, red-emitting CsMg_*x*_Pb_1−*x*_I_3_ PQDs in gadolinium-conjugated micelles efficiently produce cytotoxic ROS under 671 nm laser illumination, achieving >99% inactivation efficiency within 30 minutes post-detection, with excellent biocompatibility up to 450 ppm.^97^

For PTT, (NH_4_)_*x*_Cs_1−*x*_PbBr_3_ PQDs conjugated with IR780 dye *via* poly(styrene-*co*-maleic anhydride) exhibit a photothermal conversion efficiency of 57.85%, inducing hyperthermia in HeLa, B16F1, and HepG2 cancer cells upon laser irradiation. Uptake occurs *via* energy-dependent caveolin-mediated endocytosis, with high fluorescence brightness and good biocompatibility.^107^ Defect-passivated CsPbBr_3_ PQDs in polydopamine nanoparticles functionalized with folic acid demonstrate 41.5% PTT efficiency at 808 nm, stimulating Mn^2+^ and S^2−^ release for synergistic therapies.^98^ These light-activated therapies showcase PQDs' potential for minimally invasive, precise tumor ablation.

### 4.5. Advanced therapeutic modalities

Beyond PDT and PTT, PQDs enable chemodynamic therapy (CDT), gas therapy (GT), and drug delivery through ion release and cargo loading in tumor microenvironments. In CDT/GT, CsPbBr_3_ PQDs in manganese-enriched polydopamine with folic acid generate ·OH radicals from Mn^2+^ and H_2_S from S^2−^ under acidic conditions, blocking intracellular glutathione for enhanced CDT efficiency. Combined with PTT, this multifunctional system shows significant effectiveness against HeLa and 4T1 cells upon 808 nm laser and H_2_O_2_, with notable *in vivo* tumor accumulation and suppression.^98^

Drug delivery leverages PQDs' high surface-to-volume ratio; functionalized PQDs with lipid, protein, or inorganic modifications enable targeted chemotherapy and immunotherapy.^108,109^ Lead-free PQDs like phenanthroline-capped Cs_3_Cu_2_Cl_5_ exhibit a PLQY of 31.07% and detect tebuconazole with an LOD of 3.44 nM, suggesting safer platforms for cancer drug delivery.^110^ These modalities expand PQDs' therapeutic arsenal, integrating ROS production, gas release, and controlled delivery for comprehensive cancer treatment.

### 4.6. Integrated theranostic platforms

Theranostic platforms merge PQDs' imaging and therapeutic functions for personalized medicine, enabling real-time monitoring and treatment. Defect-passivated CsPbBr_3_ PQDs in polydopamine/FA nanospheres provide fluorescence imaging, PTT (41.5% efficiency), CDT (·OH radicals), and GT (H_2_S), internalized *via* CD44 receptors for bright cellular images. Intravenous administration yields tumor accumulation and enhanced suppression *in vivo* through hyperthermia-triggered therapies.^98^ CsMg_*x*_Pb_1−*x*_I_3_ PQDs in Pluronic F127-Gd micelles offer fluorescence-MRI dual imaging (*r*_2_/*r*_1_ 1.38) and PDT *via* ROS, with caveolae-mediated uptake.^97^. (NH_4_)_*x*_Cs_1−*x*_PbBr_3_-IR780 conjugates combine high PLQY imaging and PTT (57.85% efficiency).^107^ Stabilized PQDs like CsPbBr_3_ enable personalized strategies in treating triple-negative breast cancer (TNBC) through imaging and therapy.^93^ These platforms demonstrate PQDs' efficacy in unified systems, improving outcomes through targeted, monitored interventions.

**Fig. 5:**
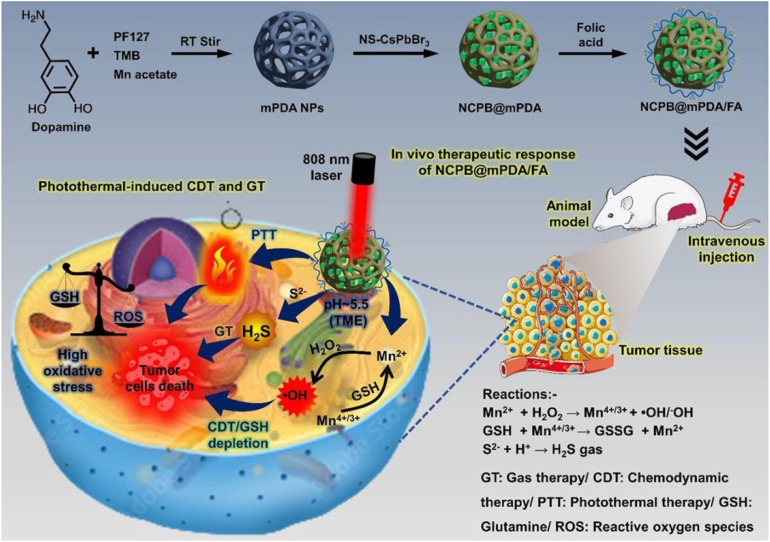
Overview of the NCPB@mPDA/FA theranostic platform. Defect-passivated CsPbBr_3_ QDs embedded in Mn-enriched polydopamine enable fluorescence imaging, photothermal heating, and pH-activated CDT/GT through Mn^2+^-mediated ·OH generation and H_2_S release. FA functionalization supports CD44-targeted uptake and enhanced *in vivo* tumor suppression. Reprinted with permission from ref. 98. Copyright (2023), Elsevier.

Within integrated theranostic systems, the NCPB@mPDA/FA platform exemplifies how multiple imaging and therapeutic modalities can be unified in a single PQD-based nanostructure. In this design, CsPbBr_3_ QDs are stabilized using a diammonium sulfide additive and subsequently embedded into Mn-enriched porous polydopamine, which not only enhances their aqueous durability but also creates a microenvironment capable of releasing Mn^2+^ and S^2−^ ions under acidic tumor conditions (Fig. 5). These ions drive chemodynamic therapy through ·OH radical generation and enable gas therapy *via* H_2_S release. Concurrently, the mPDA matrix promotes glutathione depletion, heightening oxidative stress and strengthening CDT efficiency.

Under 808 nm irradiation, the system exhibits robust photothermal activity while simultaneously accelerating ion release, thereby establishing a synergistic PTT + CDT + GT therapeutic pathway. Folic-acid functionalization further mediates CD44-dependent internalization, resulting in bright intracellular fluorescence. Following intravenous administration, the nanospheres demonstrate pronounced tumor accumulation, where the coupling of hyperthermia with CDT and GT leads to enhanced tumor suppression *in vivo*. These findings highlight, for the first time, that stabilized perovskite QDs can operate as comprehensive theranostic agents—integrating imaging, photothermal activation, and chemical/gas-mediated therapy within a single platform, advancing the development of next-generation personalized treatment strategies.

### 4.7. Case studies, emerging innovations, and future directions

Case studies illustrate PQDs' clinical potential across cancer types. In pancreatic cancer, CsPbBr_3_@SiO_2_-antibody systems enable X-ray detection of 5 mm tumors with low-dose nanoparticles. Lung cancer biosensing detects CEA/NSE with low LODs in mPADs. Breast cancer exosome imaging uses stable PQDs, while TNBC theranostics leverage water-dispersible PQDs. Gastric cancer targeting identifies xenografts with AZI-PQDs. Myeloma miRNA-155 detection achieves 73.5 pM LOD. Liver AFP sensing shows broad linear range. Emerging innovations include lead-free PQDs for safer applications and scintillation for radiation therapy monitoring. Future directions involve green synthesis, clinical translation, and integration with machine learning for optimized cancer management (Table 6).

**Table 6:** Performance metrics of PQD systems in cancer applications

Application	PQD system	Key metric	Cancer type	Ref.
Fluorescence imaging	CsPbBr_3_-AZI-CD44v6	Low toxicity; identifies xenografts	Gastric	88
X-ray imaging	CsPbBr_3_@SiO_2_-Ab	Detects 5 mm tumors; 2.8 µg dose	Pancreatic	95 and 96
Biomarker detection	CsPbI_3_/CsPbBr_3_-Ab	LOD 0.095/30 ng mL^−1^ (CEA/NSE)	Lung	102
Exosome imaging	CsPbBr_3_-polymer-anti-CD63	PLQY 88%; stable 8 days in water	Breast	89
miRNA detection	UCNPs@SiO_2_-PQDs	LOD 73.5 pM; FRET 70.6%	Myeloma	100
PEC sensing	CsPbCl_3_/TiO_2_	LOD 30 pg mL^−1^; range 0.08–980 ng mL^−1^	Liver	99
Chemiluminescence IA	CsPbBr_3_-MoS_2_-parylene	Detects AFP/HBsAg/HIV-Ab	Liver/general	103
Theranostics	CsPbBr_3_@mPDA/FA	PTT efficiency 41.5%; tumor suppression	General (HeLa/4T1)	98
PTT	(NH_4_)_*x*_Cs_1−*x*_PbBr_3_-IR780	Conversion 57.85%; biocompatibility	General	107
Dual-modal Imaging/PDT	CsMg_*x*_Pb_1−*x*_I_3_@PF127-Gd	*r*_2_/*r*_1_ 1.38; ROS generation	General	97
GSH sensing	PQD@SiO_2_-MnO_2_	Dual-mode detection; smartphone-assisted	General (oxidative stress)	105
Scintillation	CsPbBr_3_ nanocrystals	Size-dependent efficiency; reduced auger decay	Radiation therapy monitoring	101

## 5. Challenges, toxicity, and future perspectives

PQDs have shown remarkable potential in cancer diagnostics and therapeutics, leveraging their high PLQYs, tunable optical properties, and compatibility with multimodal platforms. However, their clinical translation faces significant hurdles, including instability, toxicity, and scalability issues. This section explores key challenges in deploying PQDs for clinical applications, strategies to mitigate toxicity and environmental concerns, and future research directions to enhance their biomedical utility, drawing directly from experimental and review data provided in the referenced studies.

### 5.1. Major barriers and regulatory considerations for clinical translation

This complete absence of clinical translation—in stark contrast to CdSe/ZnS or InP quantum dots, several of which have reached Phase I/II—stems from four tightly interlinked obstacles: (i) rapid physicochemical degradation in biological environments, (ii) potential heavy-metal (especially Pb^2+^) toxicity and stringent regulatory limits, (iii) lack of reproducible, GMP-compliant, large-scale manufacturing, and (iv) insufficient long-term preclinical safety data and absence of specific regulatory guidance for this emerging nanomaterial class.

The most immediate technical barrier is the extreme instability of PQDs in aqueous and physiological media. Unprotected lead-halide PQDs (*e.g.*, CsPbBr_3_, CsPbI_3_) undergo rapid hydrolysis, anion exchange, and surface-ligand detachment when exposed to water, PBS, or cell-culture media, typically losing >90% of their photoluminescence within hours.^89,91^ This degradation arises from the highly ionic character of the perovskite lattice and the relatively weak coordination of conventional oleic acid/oleylamine ligands, which are readily displaced by polar molecules. Even state-of-the-art dual-polymer encapsulation (PS-PEB-PS inner layer + PEG-PPG-PEG outer layer)—while preserving the cubic phase and retaining approximately 60% of initial PL intensity after one month in pure water (Fig. 4(a and b))—still exhibits gradual quenching in serum-containing media over weeks due to protein corona formation and slow ion penetration.^89^ Such multi-step, heterogeneous encapsulation strategies significantly increase production complexity, cost, and batch-to-batch variability, making them extremely difficult to implement under strict GMP conditions required for clinical-grade materials.

The second and arguably most decisive regulatory obstacle is the toxicity of lead-based compositions. Both the U.S. FDA and European Medicines Agency (EMA) classify nanomaterials containing Pb, Cd, or Hg as high-risk materials under ICH Q3D and relevant nanomedicine guidance documents. Regulatory acceptance demands rigorous proof that metal-ion release remains below 0.1 ppm in simulated biological fluids over at least 30 days, coupled with chronic toxicology studies (6–12 months) in two relevant species and full biodistribution/excretion profiling. Bare or insufficiently passivated CsPbBr_3_ PQDs routinely exceed these limits by orders of magnitude, exhibiting clear dose- and time-dependent cytotoxicity, ROS generation, and accumulation in liver, spleen, and kidneys.^97,108,109^ Although advanced silica, PMMA, polydopamine, or MOF shells can reduce Pb^2+^ leakage to acceptable short-term levels and improve cell viability from <70% to >95%,^90,95^ long-term *in vivo* fate, biodegradability, and complete clearance studies remain scarce or incomplete.^111^ In sharp contrast, genuinely lead-free (*e.g.*, Cs_3_Cu_2_Cl_5_, Cs_2_AgInCl_6_ derivatives) and lead-reduced (*e.g.*, Mg-doped CsMg_*x*_Pb_1−*x*_I_3_) compositions inherently satisfy heavy-metal restrictions from the outset and are therefore viewed far more favorably by regulators.^97,110^

Scalability and GMP-compliant manufacturing constitute the third critical bottleneck. Conventional hot-injection and ligand-assisted reprecipitation techniques rely on sub-second nucleation bursts, rendering precise control of size distribution, PLQY, and surface chemistry extremely sensitive to minor fluctuations in temperature, injection rate, or ligand ratio. Achieving the <5% batch-to-batch variation demanded by regulatory authorities is challenging beyond gram-scale quantities using standard laboratory reactors.^91,109^ Although continuous-flow microfluidic platforms and automated droplet systems have demonstrated kilogram-scale production with excellent monodispersity and reproducibility, no certified GMP facility has yet produced clinical-grade PQDs for IND or CTA submission. Future clinical lots will almost certainly be required to meet stringent quality attributes, including particle size PDI < 0.1, PLQY > 70% after terminal sterilization, endotoxin levels < 5 EU mL^−1^, residual organic solvents and ligands <10 ppm, and proven sterility—the latter being particularly problematic given the ionic lattice's sensitivity to gamma irradiation and inability to withstand standard filter sterilization without aggregation.

**Fig. 6:**
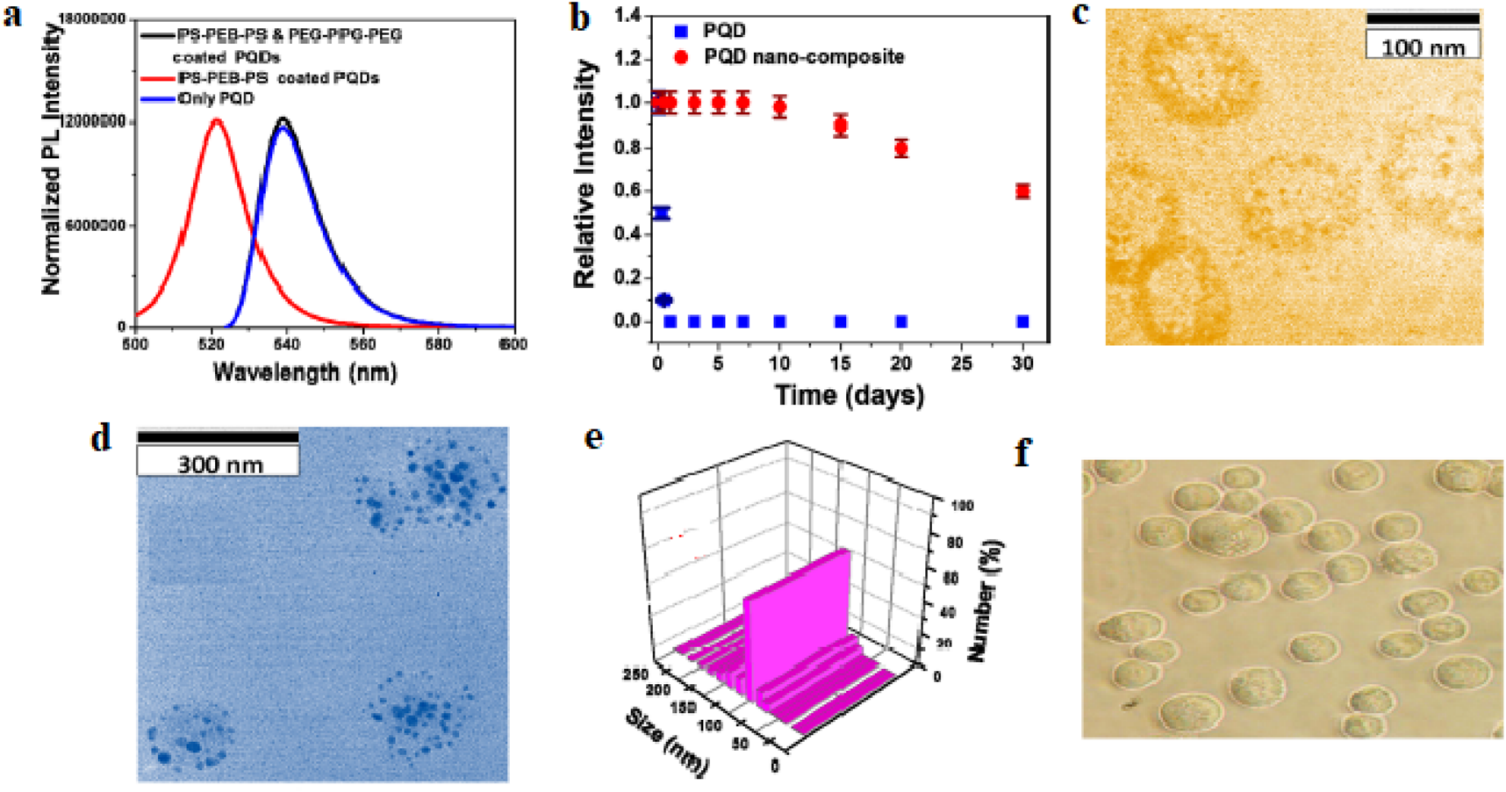
(a) Normalized PL spectra of CsPbBr_3_ PQDs in hexane, PS-PEB-PS coated, and PS-PEB-PS/PEG-PPG-PEG coated in water. (b) PL intensity *vs.* time for PQDs and nanocomposites, showing 60% retention after one month. (c) TEM of fresh TNBC-derived exosomes. (d) TEM of anti-CD63 antibody-attached PQD-conjugated exosomes. (e) DLS size distribution of exosomes. (f) Bright-field image of HaCaT cells without nanocomposite binding. Adapted with permission from ref. 89, Copyright 2019 American Chemical Society.

Fig. 6 exemplifies both the severity of the aqueous instability problem and a promising engineering solution. Panels (a) and (b) compare normalized PL spectra and long-term intensity retention of bare CsPbBr_3_ PQDs *versus* dual-polymer-encapsulated nanocomposites in water, clearly showing complete quenching of unprotected dots within days while the PS-PEB-PS/PEG-PPG-PEG coating preserves ∼60% emission after one month. Panels (c) and (e) present TEM and DLS characterization of triple-negative breast cancer (TNBC)-derived exosomes (150 ± 50 nm, CD63-positive), establishing a clinically relevant targeting model. Panels (d) and (f) demonstrate highly selective binding of anti-CD63-functionalized PQD nanocomposites to exosomes (visible surface attachment) but not to normal HaCaT cells (no binding in bright-field imaging), proving that sophisticated surface engineering can simultaneously confer aqueous stability, colloidal robustness, and tumor-specific recognition—a critical combination for future clinical translation. Until robust, GMP-compatible encapsulation protocols or fully lead-free compositions are paired with comprehensive chronic toxicology packages and formal pre-IND/CTA regulatory consultations, clinical advancement of PQDs will remain blocked. Lead-free and lead-minimized variants currently offer by far the clearest and most realistic path toward regulatory acceptance and first-in-human studies.

### 5.2. Innovative mitigation of toxicity and environmental concerns

To address toxicity and environmental challenges, innovative encapsulation and compositional strategies have been developed to enhance PQD stability and safety. Polymer encapsulation is a widely adopted approach to prevent Pb^2+^ leakage and improve aqueous stability. For example, CsPbBr_3_ PQDs encapsulated in polystyrene-*block*-poly(ethylene-*ran*-butylene)-*block*-polystyrene (PS-PEB-PS) and poly(ethylene glycol)-*block*-poly(propylene glycol)-*block*-poly(ethylene glycol) (PEG-PPG-PEG) exhibit improved PLQY (from 83% to 88%) and retain luminescence in water for 8 days, with only a 40% intensity drop after one month, compared to complete quenching in unencapsulated PQDs.^89^ Similarly, PMMA-encapsulated CsPbBr_3_ PQDs used for miRNA-21 imaging show negligible cytotoxicity, attributed to reduced ion leakage.^90^ Silica-based encapsulation is another effective strategy. Double-encapsulated CsPbBr_3_ PQDs in SiO_2_ shells prevent aggregation, decomposition, and Pb^2+^ release, enabling safe X-ray imaging of pancreatic tumors with confirmed biocompatibility *via* cell viability and histological assays.^95^ Silica-coated PQDs at the single-particle level, combined with MnO_2_ quenchers, further enhance stability for glutathione sensing, relevant to cancer oxidative stress, with no reported toxicity. These silica shells also improve environmental safety by reducing heavy metal leaching into ecosystems.^111^

Surface passivation techniques address both stability and toxicity. For instance, (NH_4_)_2_S-treated CsPbBr_3_ PQDs show superior optical properties and aqueous stability when loaded into manganese-enriched polydopamine nanoparticles, functionalized with folic acid for cancer theranostics. These systems exhibit biocompatibility up to 450 ppm, with no significant toxicity in HeLa and 4T1 cells.^98^ Ligand engineering, such as d-tartaric acid functionalization of CsPbBr_3_ PQDs, yields a PLQY of 29.88% and stable emission in aqueous media, suitable for bio-imaging with minimal environmental impact. Lead-free PQDs offer a promising solution to toxicity concerns. Phenanthroline-capped Cs_3_Cu_2_Cl_5_ PQDs, with a PLQY of 31.07%, demonstrate high stability and low toxicity, making them viable for sensing applications with potential extension to cancer drug delivery. Replacing Pb^2+^ with Mg^2+^ in CsMg_*x*_Pb_1−*x*_I_3_ PQDs enhances PLQY to 89% and stability, with no significant cytotoxicity observed *in vivo*.^97^ These strategies—polymer and silica encapsulation, surface passivation, and lead-free compositions—collectively reduce toxicity and environmental risks, paving the way for safer clinical applications.

### 5.3. Future directions: novel research avenues and technological innovations

Future research on PQDs in cancer applications focuses on overcoming current limitations through novel synthesis methods, advanced functionalization, and integration with emerging technologies. Developing green synthesis techniques is a priority to enhance scalability and reduce environmental impact. Room-temperature synthesis of monodisperse APbBr_3_ PQDs (A = Cs, formamidinium, methylammonium) with controlled sizes from 3 to >13 nm demonstrates improved reproducibility, addressing scalability challenges.

Advanced functionalization strategies aim to enhance specificity and stability. Conjugating PQDs with cancer-specific ligands, such as antibodies or peptides, can improve targeting precision. For example, AZI-modified CsPbBr_3_ PQDs with CD44v6 peptides achieve specific gastric cancer imaging, suggesting a model for ligand-driven targeting in other cancers.^88^ Reviews propose integrating PQDs with biomolecules like aptamers or nucleic acids for enhanced selectivity in diagnostics and drug delivery. Molecularly imprinted polymers (MIPs), as demonstrated with MIP@MAPbBr_3_ PQDs for benzo(*a*)pyrene detection (LOD 1.6 ng mL^−1^), offer a template for developing highly selective cancer biomarker sensors.^104^

Integration with machine learning (ML) presents a transformative avenue. ML-driven CsPbBr_3_ PQD sensors achieve 100% accuracy in identifying pathogens, with potential adaptation for cancer biomarker profiling, enhancing diagnostic precision.^106^ Smartphone-assisted platforms, such as those for glutathione sensing with silica-coated PQDs, enable portable, cost-effective diagnostics, suggesting future applications in point-of-care cancer screening. Lead-free and double perovskite PQDs are critical for clinical translation. Cs_3_Cu_2_Cl_5_ PQDs and other non-toxic variants offer safer alternatives for *in vivo* applications, with ongoing research into their optical tunability for imaging and therapy. Stabilized PQDs for triple-negative breast cancer (TNBC) imaging and therapy highlight the potential of water-dispersible, lead-free systems.^93^

Multimodal theranostic platforms combining imaging, PDT, PTT, and drug delivery are a key focus. Systems like CsPbBr_3_@polydopamine/FA, integrating fluorescence, MRI, PTT (41.5% efficiency), CDT, and GT, demonstrate tumor suppression *in vivo* and serve as a blueprint for future innovations.^98^ Scintillation-based PQDs, with size-dependent efficiency, offer potential for real-time radiation therapy monitoring, improving treatment outcomes.

Clinical translation requires addressing regulatory and safety concerns through long-term *in vivo* studies. Current data, such as biocompatibility of CsPbBr_3_@SiO_2_ in pancreatic tumor imaging,^95^ provide a foundation, but extensive toxicological profiling is needed. Collaborative efforts between material scientists, oncologists, and regulatory bodies will be crucial to establish PQDs as viable clinical tools. In summary, overcoming stability and toxicity challenges through advanced encapsulation, lead-free compositions, and green synthesis, combined with innovations in functionalization and ML integration will drive PQDs toward clinical success in cancer diagnostics and therapeutics (Table 7).

**Table 7:** Strategies to address PQD challenges in cancer applications

Challenge	Mitigation strategy	Example	Ref.
Aqueous instability	Polymer encapsulation	CsPbBr_3_@PS-PEB-PS/PEG-PPG-PEG, 8-day stability	89
Toxicity	Silica encapsulation	CsPbBr_3_@SiO_2_, nontoxic *in vivo*	95 and 96
Toxicity	Surface passivation	(NH_4_)_2_S-treated CsPbBr_3_, biocompatible up to 450 ppm	98
Toxicity	Lead-free PQDs	Cs_3_Cu_2_Cl_5_, PLQY 31.07%	110
Scalability	Green synthesis	Room-temperature APbBr_3_ synthesis, 3–13 nm	112
Specificity	Ligand conjugation	AZI-PQDs-CD44v6 for gastric cancer	88
Regulatory	Long-term studies	Needed for CsPbBr_3_ biocompatibility	92

### 5.4. Degradation products, long-term biodistribution, and clearance of lead-based PQDs

Even with the most advanced encapsulation strategies discussed in Section 3, no protective shell is indefinitely impermeable. The eventual *in vivo* fate of lead-based PQDs is therefore determined by the nature and biological handling of their degradation products, primarily Pb^2+^ ions, halide anions (Br^−^/I^−^), and residual organic ligands released through gradual hydrolysis, lysosomal degradation, or enzymatic attack.^23,89,91^ In uncoated CsPbBr_3_ PQDs, moisture-induced lattice dissociation can liberate 10–30% of total lead within 24–72 h in physiological media (pH 4.5–7.4, 37 °C).^89,91^ State-of-the-art dual-polymer (PS-PEB-PS/PEG-PPG-PEG) or silica coatings reduce Pb^2+^ release to below 0.1 ppm over 30 days—meeting FDA parenteral impurity thresholds—but long-term exposure still results in slow ion leakage as micro-cracks form under continuous biological stress.^89,95^

Once released, free Pb^2+^ rapidly binds serum proteins and intracellular thiols, forming stable complexes that dramatically prolong systemic retention compared with intact nanoparticles.^97,108^ Biodistribution studies of intravenously administered coated CsPbBr_3_ PQDs consistently demonstrate predominant accumulation in the reticuloendothelial system: liver uptake reaches 30–45% injected dose per gram at 24 h and remains >15% ID g^−1^ after 28 days, with substantial splenic retention (10–25% ID g^−1^).^97,109^ Renal clearance of intact nanoparticles (>6–8 nm) is limited (<5% ID in urine within 7 days), while hepatobiliary excretion accounts for only 30–50% ID over 14–30 days, depending on PEG density and surface charge.^55,90^ Critically, protein-bound Pb^2+^ exhibits far slower elimination kinetics than the nanoparticulate fraction, with detectable lead persisting in liver, spleen, and brain beyond 90 days in several reports, raising serious concerns regarding cumulative neurotoxicity, oxidative stress, and genotoxicity.^108,109^

Current mitigation strategies remain insufficient for clinical acceptance. Although robust silica or PMMA shells significantly suppress acute toxicity,^90,95^ they do not guarantee complete long-term containment or rapid excretion of degradation products. Biodegradable polymer matrices and stimuli-responsive linkers have been explored to promote controlled disassembly and faster renal filtration of sub-6 nm fragments,^57^ but these approaches often compromise initial stability or brightness. Consequently, as emphasized throughout Sections 3.5 and 5.1, genuinely lead-free compositions (*e.g.*, Cs_3_Cu_2_Cl_5_, Mg-doped CsMg_*x*_Pb_1−*x*_I_3_) that eliminate heavy-metal degradation products from the outset currently represent the only realistic pathway capable of satisfying stringent regulatory requirements for chronic safety, biodistribution, and clearance.^97,110^ Until fully validated 6–12-month excretion data become available for lead-based systems, their clinical translation will remain effectively blocked.

## 6. Conclusion

PQDs have emerged as a transformative tool in cancer diagnostics and therapeutics, offering innovative solutions for early detection and targeted treatment. Their unique properties enable high-sensitivity imaging and biosensing, significantly advancing oncology. Fluorescence-based systems provide clear visualization of gastric cancer cells and xenografts with minimal toxicity, supporting real-time tumor monitoring. X-ray imaging detects small pancreatic tumors using low nanoparticle doses, ensuring safety through biocompatibility. Paper-based microfluidic devices identify lung cancer biomarkers with high precision, while photoelectrochemical sensors target liver cancer markers across a broad detection range. Upconversion-modulated platforms pinpoint myeloma biomarkers, facilitating early diagnosis and intervention.

Therapeutically, PQDs excel in delivering precise cancer treatments. Photodynamic therapy generates reactive oxygen species to induce tumor cell death, while photothermal therapy delivers localized heat to eliminate cancer cells effectively. Multifunctional systems combine chemodynamic therapy, producing hydroxyl radicals, and gas therapy, releasing therapeutic gases in tumor microenvironments, achieving robust tumor suppression. PQDs also enhance drug delivery by conjugating with cancer-specific ligands, improving chemotherapy and immunotherapy outcomes. Theranostic platforms integrate imaging and treatment, enabling personalized medicine through real-time monitoring and tailored interventions. Despite these advancements, challenges such as aqueous instability, potential toxicity, and scalability persist. Encapsulation with polymers or silica, surface passivation, and lead-free compositions have improved stability and safety, reducing environmental and health risks. Advances in scalable synthesis methods further support clinical translation. By addressing these hurdles through innovative materials and integration with technologies like machine learning, PQDs hold immense promise for revolutionizing cancer care, offering precise diagnostics and effective therapies to improve patient outcomes.

## Author contributions

M. Abushuhel and H. Noorizadeh conceived the idea and supervised the project. M. Abushuhel, G. PadmaPriya, S. Al-Hasnaawei, K. Chennakesavulu and M. Kazemi wrote the original draft of the manuscript. All authors (M. Abushuhel, G. PadmaPriya, S. Al-Hasnaawei, S. Ray, K. Chennakesavulu, R. Sharma, A. S. Chauhan, H. Noorizadeh and M. Kazemi) contributed to investigation, data curation, validation, writing – review & editing, and visualization of the review content. All authors have read and agreed to the final version of the manuscript.

## Conflicts of interest

There are no conflicts to declare.

## Data Availability

No primary research results, software or code have been included and no new data were generated or analysed as part of this review.

## References

[cit1] Xiong X., Zheng L. W., Ding Y., Chen Y. F., Cai Y. W., Wang L. P., Huang L., Liu C. C., Shao Z. M., Yu K. D. (2025). Signal Transduction Targeted Ther..

[cit2] Kureshi C. T., Dougan S. K. (2025). Cancer Cell.

[cit3] Filho A. M., Laversanne M., Ferlay J., Colombet M., Piñeros M., Znaor A., Parkin D. M., Soerjomataram I., Bray F. (2025). Int. J. Cancer.

[cit4] Montégut L., López-Otín C., Kroemer G. (2024). Mol. Cancer.

[cit5] Zafar A., Khatoon S., Khan M. J., Abu J., Naeem A. (2025). Discover Oncol..

[cit6] Dash S. R., Kundu A., Kundu C. N. (2024). Life Sci..

[cit7] Webster M., Podgorsak A., Li F., Zhou Y., Jung H., Yoon J., Lemus O. D., Zheng D. (2025). Cancers.

[cit8] Zhu N., Ni H., Guo S., Shen Y. Q., Chen Q. (2024). Cancer Treat. Rev..

[cit9] Joshi R. M., Telang B., Soni G., Khalife A. (2024). Oncol. Transl. Med..

[cit10] Alsaikhan F., Farhood B. (2024). Int. J. Biol. Macromol..

[cit11] Liu B., Zhou H., Tan L., Siu K. T., Guan X. Y. (2024). Signal Transduction Targeted Ther..

[cit12] Rasool S., Ali M., Shahroz H. M., Hussain H. K., Gill A. Y. (2024). J. Multidiscip. Sci..

[cit13] Yang T., Guo L. (2024). Cell Biol. Toxicol..

[cit14] Sun L., Lan J., Li Z., Zeng R., Shen Y., Zhang T., Ding Y. (2024). Int. J. Nanomed..

[cit15] Maleki H., Aiyelabegan H. T., Javadi P., Abdi F., Mirzavi F., Behjani Z. Z., Rizvanov A. A., Takallu S., Kumar R., Barhaghtalab R. H., Hosseinpouri A. (2025). Biomed. Pharmacother..

[cit16] Osorio H. M., Castillo-Solís F., Barragán S. Y., Rodríguez-Pólit C. (2024). Int. J. Mol. Sci..

[cit17] Noorizadeh H. (2025). Chem. Pharm. Lett..

[cit18] Karthik A., Aalam S. S., Sivakumar M., Sundari M. R., Rose J. D., Elangovan M., Rajaram A. (2024). Biomed. Signal Process. Control.

[cit19] Sarkar S., Srivastava T. P., Sahoo O. S., Shankar A., Rai A., Pethusamy K., Dhar R., Karmakar S. (2024). Asian Pac. J. Cancer Prev..

[cit20] Chen L., Jiang C., Scholle F., Meo A. E., Ohata J., Gorman C. B., Ghiladi R. A. (2025). ACS Appl. Bio Mater..

[cit21] Wang Q., Xue X., Wang P., Yu Y., Wang J., Jiang Q., Xiao J. (2024). Front. Pharmacol.

[cit22] Wu X. G., Jing Y., Zhong H. (2025). Adv. Mater..

[cit23] Altoum A., Abbood R. S., Abbas al-Khafaji Z., Kachhiya P., Menon S. V., Sunitha S., Singla S., Hamzah H. F., Mustafa Y. F., Noorizadeh H. (2025). Mater. Technol..

[cit24] Kim D., Yun T., An S., Lee C. L. (2024). Nano Convergence.

[cit25] Shan Q., Dong Y., Xiang H., Yan D., Hu T., Yuan B., Zhu H., Wang Y., Zeng H. (2024). Adv. Funct. Mater..

[cit26] Xu B., Yuan S., Wang L., Li X., Hu Z., Zeng H. (2025). ACS Nano.

[cit27] Mi C., Gee G. C., Lander C. W., Shin D., Atteberry M. L., Akhmedov N. G., Hidayatova L., DiCenso J. D., Yip W. T., Chen B., Shao Y. (2025). Nat. Commun..

[cit28] Chen J., Chen S., Liu X., Zhu D., Cai B., Luo X., Feng W., Cheng Y., Xiong Y., Du J., Li Z. (2025). Sci. Adv..

[cit29] Zhang X., Huang H., Zhao C., Yuan J. (2025). Chem. Soc. Rev..

[cit30] Zhang L., Wang C., Zhan C. (2025). Mater. Chem. Front..

[cit31] Bai Y., Hao M., Ding S., Chen P., Wang L. (2022). Adv. Mater..

[cit32] Liu L., Najar A., Wang K., Du M., Liu S. (2022). Adv. Sci..

[cit33] Korde V. B., Khot S., Kamble D. B., Amalraj S. (2024). Sens. Actuator Rep..

[cit34] Sanjayan C. G., Ravikumar M. S., Balakrishna R. G. (2022). J. Mater. Chem. C.

[cit35] Deng Y., Yuan Y., Ni J., Zheng L., Bi J., Guo J., Wang R., Li H., Zhang S., Cai J. (2025). ACS Appl. Energy Mater..

[cit36] Chi W., Banerjee S. K. (2022). Angew. Chem., Int. Ed..

[cit37] Hao M., Ding S., Gaznaghi S., Cheng H., Wang L. (2024). ACS Energy Lett..

[cit38] Tu S., Chen M., Wu L. (2021). Chem. Eng. J..

[cit39] Ye J., Gaur D., Mi C., Chen Z., Fernández I. L., Zhao H., Dong Y., Polavarapu L., Hoye R. L. (2024). Chem. Soc. Rev..

[cit40] Zhang P., Yang G., Li F., Shi J., Zhong H. (2022). Nat. Commun..

[cit41] Zhang X., Li L., Chen Y., Valenzuela C., Liu Y., Yang Y., Feng Y., Feng W. (2024). Angew. Chem..

[cit42] Zou J., Li M., Zhang X., Zheng W. (2022). J. Appl. Phys..

[cit43] Liu Y., Li Y., Hu X., Wei C., Xu B., Leng J., Miao H., Zeng H., Li X. (2023). Chem. Eng. J..

[cit44] Peighambardoust N. S., Sadeghi E., Aydemir U. (2022). ACS Appl. Nano Mater..

[cit45] Ren X., Zhang X., Xie H., Cai J., Wang C., Chen E., Xu S., Ye Y., Sun J., Yan Q., Guo T. (2022). Nanomaterials.

[cit46] He H., Mei S., Wen Z., Yang D., Yang B., Zhang W., Xie F., Guo R. (2022). Small.

[cit47] Lim S., Han S., Kim D., Min J., Choi J., Park T. (2023). Adv. Mater..

[cit48] Aftab S., Koyyada G., Mukhtar M., Hegazy H. H., Kim J. H. (2024). J. Mater. Chem. C.

[cit49] Li J., Nong Y., Yao J., Xu L., Yang Z., Wang S., Song J. (2025). Adv. Funct. Mater..

[cit50] Han Y., Chang X., Cheng X., Lin Y., Cui B. B. (2023). Laser Photonics Rev..

[cit51] Liao S., Yang Z., Lin J., Wang S., Zhu J., Chen S., Huang F., Zheng Y., Chen D. (2023). Adv. Funct. Mater..

[cit52] Qiao G. Y., Guan D., Yuan S., Rao H., Chen X., Wang J. A., Qin J. S., Xu J. J., Yu J. (2021). J. Am. Chem. Soc..

[cit53] Wu Y., Dai S., Liu X., Guo P., Zhang J., Sun T., Guo Z., Xu Y., Liang H., Xiong L., Hu H. (2024). Adv. Funct. Mater..

[cit54] Zhang X., Huang H., Zhao C., Jin L., Lee C., Li Y., Ko C., Ma W., Wu T., Yuan J. (2024). Nat. Energy.

[cit55] Ma C., Zhang M., Zhang J., Liao J., Sun H., Ji D., Pang R., Zhang H., Liu J., Liu S. (2024). Adv. Funct. Mater..

[cit56] Xie M., Guo J., Zhang X., Bi C., Zhang L., Chu Z., Zheng W., You J., Tian J. (2022). Nano Lett..

[cit57] Tian J., Tan Q. Y., Wang Y., Yang Y., Yuan G., Adamo G., Soci C. (2023). Nat. Commun..

[cit58] Mei X., Jia D., Chen J., Zheng S., Zhang X. (2022). Nano Today.

[cit59] Kim T., Park B. (2024). Chem. Mater..

[cit60] Feld L. G., Boehme S. C., Morad V., Sahin Y., Kaul C. J., Dirin D. N., Rainò G., Kovalenko M. V. (2024). ACS Nano.

[cit61] Feld L. G., Shynkarenko Y., Krieg F., Rainò G., Kovalenko M. V. (2021). Adv. Opt. Mater..

[cit62] Sheng Y., Chen W., Hu F., Liu C., Di Y., Sheng C., Chen Z., Jia B., Wen X., Gan Z. (2022). ACS Appl. Mater. Interfaces.

[cit63] Liu B., Hu J., Liu X., Xu H., Dong Z. (2025). J. Colloid Interface Sci..

[cit64] Sharma D., Sharma S. K. (2024). Opt. Mater..

[cit65] Huang S. Q., Chung S. R. (2025). Proc. SPIE.

[cit66] Nazir G., Koyyada G., Rehman A., Hussain S., Aftab S., Khalid A., Hafez A. A., Heo K., Kim J. H. (2025). J. Alloys Compd..

[cit67] Zhan X. (2024). Appl. Comput. Eng..

[cit68] Zhang M., Huang S., Mei X., Wang G., Ren B., Qiu J., Zhang X. (2025). Energy Environ. Sci..

[cit69] Long Z., Yang S., Pi J., Zhou D., Wang Q., Yang Y., Wu H., Qiu J. (2022). Ceram. Int..

[cit70] Soosaimanickam A., Manidurai P., Sundaram S. K. (2023). Nanomaterials.

[cit71] Huang C. H., Chu S. Y. (2024). SSRN Electron. J..

[cit72] Costa W. C., Salla C. A., Ely F. (2021). Nanotechnology.

[cit73] Lee C., Lee S. J., Shin Y., Woo Y., Han S. H., Gualdrón-Reyes A. F., Mora-Sero I., Yoon S. J. (2021). Catalysts.

[cit74] Jabbar Khan A., Gao L., Numan A., Khan S., Hussain I., Sajjad M., Shah S. S., Mateen A. (2025). Crit. Rev. Solid State Mater. Sci..

[cit75] Hachem K., Ansari M. J., Saleh R. O., Kzar H. H., Al-Gazally M. E., Altimari U. S., Hussein S. A., Mohammed H. T., Hammid A. T., Kianfar E. (2022). BioNanoScience.

[cit76] Ahmed D. S., Mohammed M. K., Majeed S. M. (2020). ACS Appl. Energy Mater..

[cit77] Lozano M. S., Gómez V. J. (2023). Nanoscale Adv..

[cit78] Al-Douri Y., Khan M. M., Jennings J. R. (2023). J. Mater. Sci.: Mater. Electron..

[cit79] Liu Y., Li F., Huang H., Mao B., Liu Y., Kang Z. (2020). J. Semicond..

[cit80] Chen B., Li D., Wang F. (2020). Small.

[cit81] Rajan D., Manoj B., Krishna S., Thomas A. (2025). ACS Energy Lett..

[cit82] Guan X., Li Z., Geng X., Lei Z., Karakoti A., Wu T., Kumar P., Yi J., Vinu A. (2023). Small.

[cit83] Permatasari F. A., Irham M. A., Bisri S. Z., Iskandar F. (2021). Nanomaterials.

[cit84] Saraiva A., Lim W. H., Yang C. H., Escott C. C., Laucht A., Dzurak A. S. (2022). Adv. Funct. Mater..

[cit85] Liu Y., Sun W., Xiao J., Fu Y., Shi B., Lü C. (2022). Appl. Clay Sci..

[cit86] Vanalakar S. A., Qureshi M. H., Srivastava S. B., Khan S. U., Eren G. O., Onal A., Kaya L., Pehlivan H. N., Pehlivan Ç., Vhanalakar S. A. (2024). IEEE Trans. Biomed. Eng..

[cit87] Liu L., Hong J., Wang W., Xiao S., Xie H., Wang Q., Gan N. (2022). J. Pharm. Anal..

[cit88] Zhang D., Wang H., Chen C., Lu G., Yin Y., Ren M., Huang J. (2024). Nanotechnology.

[cit89] Pramanik A., Gates K., Patibandla S., Davis D., Begum S., Iftekhar R., Alamgir S., Paige S., Porter M. M., Ray P. C. (2019). ACS Appl. Bio Mater..

[cit90] Yang Z., Zhou J., Liu F., Chai Y., Zhang P., Yuan R. (2024). Anal. Chem..

[cit91] Zhang X., Lin J. R., Wei Y., Liu Y. F. (2025). J. Alloys Compd..

[cit92] Mohammadi J., Hheidari A., Sardari S., Nouri M., Ebrahimi S., Rahdar A., Pishbin E. (2024). Biomed. Mater..

[cit93] Yoon S. B., Hwang S., Kim Y., Kim B. G., Na H. B. (2024). Korean J. Chem. Eng..

[cit94] Velamala L. K., Patel M. R., Deshpande M. P., Gul A. R., Park T. J., Kailasa S. K. (2024). J. Mol. Liq..

[cit95] Ryu I., Ryu J. Y., Choe G., Kwon H., Park H., Cho Y. S., Du R., Yim S. (2021). Adv. Funct. Mater..

[cit96] Ryu I., Ryu J. Y., Choe G., Kwon H., Park H., Cho Y. S., Du R., Yim S. (2021). Adv. Funct. Mater..

[cit97] Getachew G., Korupalli C., Rasal A. S., Dirersa W. B., Fahmi M. Z., Chang J. Y. (2021). ACS Appl. Mater. Interfaces.

[cit98] Getachew G., Tien Y. C., Kan T. C., Dirersa W. B., Wibrianto A., Orchirbat S., Chang J., Rasal A. S., Gurav V., Kizhepat S., Chang J. Y. (2023). Chem. Eng. J..

[cit99] Qin J., Cui S., Yang X., Yang G., Zhu Y., Wang Y., Qiu D. (2019). J. Phys. D: Appl. Phys..

[cit100] He Y., Rao H., Wang J., Wu Y., Han C., Yan C., Temple H., Zhang L., Chen W., Liu Y. (2023). Cancer Nanotechnol..

[cit101] Fratelli A., Zaffalon M. L., Mazzola E., Dirin D. N., Cherniukh I., Otero-Martínez C., Salomoni M., Carulli F., Rossi F., Meinardi F., Gironi L. (2025). Adv. Mater..

[cit102] Sanjayan C. G., Ravikumar C. H., Balakrishna R. G. (2023). Chem. Eng. J..

[cit103] Kim H. R., Bong J. H., Park J. H., Song Z., Kang M. J., Son D. H., Pyun J. C. (2021). ACS Appl. Mater. Interfaces.

[cit104] Liu L., Peng M., Xu K., Xia H., Peng X., Peng L. (2023). Microchim. Acta.

[cit105] Chen J., Wu H., Zhang W., Huang Y., Zheng J., Shao J., Chi Y. (2025). Sens. Actuators, B.

[cit106] Zhang S., Zhu W., Zhang X., Mei L., Liu J., Wang F. (2025). J. Hazard. Mater..

[cit107] Getachew G., Huang W. W., Chou T. H., Rasal A. S., Chang J. Y. (2022). J. Colloid Interface Sci..

[cit108] Guo W., Song X., Liu J., Liu W., Chu X., Lei Z. (2024). Nanomaterials.

[cit109] Li M., Huang Y., Shen C., Wang Y., Lin Y. A., Wang Z., Chen N., Luo Y. (2025). Nano Res..

[cit110] Patel M. R., Chetti P., Park T. J., Kailasa S. K. (2025). ACS Appl. Nano Mater..

[cit111] Hamidu A., Pitt W. G., Husseini G. A. (2023). Nanomaterials.

[cit112] Akkerman Q. A., Nguyen T. P., Boehme S. C., Montanarella F., Dirin D. N., Wechsler P., Beiglböck F., Rainò G., Erni R., Katan C., Even J. (2022). Science.

[cit113] Yan D., Zhao S., Zhang Y., Wang H., Zang Z. (2022). Opto-Electron. Adv..

[cit114] Wang H., Zhang P., Zang Z. (2020). Appl. Phys. Lett..

[cit115] Guan H., Zhao S., Wang H., Yan D., Wang M., Zang Z. (2020). Nano Energy.

